# *In Vitro* and *In Vivo* Models of *Staphylococcus aureus* Endophthalmitis Implicate Specific Nutrients in Ocular Infection

**DOI:** 10.1371/journal.pone.0110872

**Published:** 2014-10-23

**Authors:** Ama Sadaka, Kelli Palmer, Takashi Suzuki, Michael S. Gilmore

**Affiliations:** 1 Departments of Ophthalmology, and Microbiology and Molecular Genetics, Harvard Medical School, Boston, Massachusetts, United States of America; 2 The Massachusetts Eye and Ear Infirmary, Boston, Massachusetts, United States of America; 3 Harvard Microbial Sciences Initiative, Cambridge, Massachusetts, United States of America; University of Rochester Medical Center, United States of America

## Abstract

**Purpose:**

To define global transcriptional responses of *Staphylococcus aureus* and its *codY* mutant (CodY is a transcription regulator of virulence and metabolic genes in response to branched-chain amino acids) when growing in bovine aqueous (AH) and vitreous humor (VH) *in vitro*, and to investigate the impact of *codY* deletion on *S. aureus* virulence in a novel murine anterior chamber (AC) infection model.

**Methods:**

For the *in vitro* model, differential transcriptomic gene expression of *S. aureus* and its *codY* mutant grown in chemically defined medium (CDM), AH, and VH was analyzed. Furthermore, the strains were inoculated into the AC of mice. Changes in bacterial growth, electroretinography and inflammation scores were monitored.

**Results:**

Bovine AH and VH provide sufficient nutrition for *S. aureus* growth *in vitro*. Transcriptome analysis identified 72 unique open reading frames differentially regulated ≥10-fold between CDM, AH, and VH. In the AC model, we found comparable growth of the *codY* mutant and wild type strains *in vivo*. Average inflammation scores and retinal function were significantly worse for *codY* mutant-infected eyes at 24 h post-infection.

**Conclusion:**

Our *in vitro* bovine AH and VH models identified likely nutrient sources for *S. aureus* in the ocular milieu. The *in vivo* model suggests that control of branched-chain amino acid availability has therapeutic potential in limiting *S. aureus* endophthalmitis severity.

## Introduction

*Staphylococcus aureus* is a commensal bacterium on the skin and mucosa, but is also a leading cause of infections in humans. When opportunistic pathogens infect sterile sites, they adapt, proliferate in the host, and exhibit virulence. The host becomes the sole source for nutrients. For many gram-positive bacteria, CodY provides an important regulatory link between nutrient availability and virulence factor production [1]. CodY controls expression of virulence and metabolic genes in response to the availability of branched-chain amino acids (BCAA) and GTP through Agr, a global regulator of the staphylococcal virulon [2]. In the presence of GTP and/or BCAA, CodY shows a higher affinity for its DNA targets, while in the absence of nutrients, there is a decrease in the GTP and BCAA levels causing decreased affinity of CodY to the DNA and thus induction of its regulon. In *S. aureus*, CodY regulates its regulon either indirectly via the quorum sensing regulator Agr or independent of the Agr system and through its direct binding to its DNA targets [2]. In general, CodY-regulated genes trigger adaptation to starvation [2]–[9] as well as play a role in virulence of pathogenic bacteria [3], [4], [10].

The human eye as well as eyes of animals like rabbits and mice possess sterile anterior and posterior compartments, which contain aqueous and vitreous humor, respectively. Infectious endophthalmitis [inflammation within the eye) is a complication of penetrating trauma to the eye and intraocular procedures such as cataract surgery that can lead to blindness [11]–[14]. The visual prognosis following infection depends greatly on the virulence of the causative organism, visual acuity at presentation, and the efficacy of antimicrobial treatment [15]. *S. aureus* is the second most common cause of acute postoperative infection following intraocular surgeries and is also associated with significant visual loss [15]. Given the presence of BCAA in human aqueous and vitreous humors [16], [17] and the findings that CodY controls directly and indirectly *S. aureus* virulence genes such as *hla* and *agr* [1], [2] which contribute to virulence in animal models of endophthalmitis [18], [19], CodY may play a role in regulating *S. aureus* virulence during endophthalmitis.

*S. aureus* growth and virulence in animal models of endophthalmitis has been assessed, most frequently by intravitreal (posterior chamber) injection [18], [19]. *S. aureus* strains grow *in vivo* to different extents, depending on the strain used and the inoculation site (anterior versus posterior compartments) [20], [21]. For example, following injection into the anterior chamber, Balzli, et al. found that among 9 *S. aureus* isolates injected into the anterior chamber of rabbit eyes, only one strain, UMCR1, grew [20]. Wu, et al. [21] and Kowalski, et al. [22] found that *S. aureus* grew in the anterior chamber of rabbits, and used that model to test antibiotic efficacies. Several other studies have found that *S. aureus* can grow to high densities in the vitreous, and studied the contribution of toxins, the global virulence regulators Agr and Sar [18], [23], and other cell wall components to pathogenesis. *S. aureus* clearly survives in the human eye, given that *S. aureus* can be recovered from the aqueous and vitreous humors of patients who develop endophthalmitis [24]. It is unknown what nutrient sources *S. aureus* utilizes during infection of the human eye. In this study, we use aqueous and vitreous fluids extracted from commercially-obtained bovine eyes as *ex vivo* endophthalmitis models for *S. aureus*, and define global transcriptional responses of *S. aureus* to growth in these media. Our goal was to identify genes that are consistently and highly differentially regulated by *S. aureus* during growth in pooled bovine AH and VH samples. We additionally interrogate the impact of *codY* deletion on *S. aureus* gene expression during growth in these media, as well as its impact on *S. aureus* virulence in a novel murine anterior chamber (AC) infection model.

## Materials and Methods

### Strains and growth media

*S. aureus* strains used are listed in Table 1. *S. aureus* was routinely cultured in brain heart infusion (BHI) or on BHI agar. All cultures were incubated at 37°C. For microarray experiments, *S. aureus* were grown in chemically defined Socransky's medium [25] supplemented with 20 mM glucose (referred to here as CDM), or bovine aqueous or vitreous humor (AH and VH, respectively). CDM contains 76 µM leucine, 85 µM valine, and 76 µM isoleucine. Bacterial growth was assessed by monitoring optical density at 600 nm (OD_600_) using a Biotech Synergy 2 microplate reader or by serial dilution and plating on BHI agar to obtain colony forming units per milliliter (CFU/mL).

**Table 1:** Bacterial strains used in this study.

*S. aureus* strain	Description	Reference/source
SA564	Clinical isolate	[48]
CDM7	SA564 Δ*codY::ermC*	[29]
MS7	SA564 Δ*codY::ermC* pTL6936-*codY*	[29]

### Bovine AH and VH collection

AH and VH were extracted from commercially available bovine eyes (Sierra for Medical Science, Whittier, CA) and pooled as described previously [26]. Typical volumes of AH and VH recovered per bovine eye were 0.5–1 mL and 3–4 mL, respectively. AH was filter-sterilized with a 0.45 µm HT Tuffryn membrane sterile acrodisc syringe filter (Pall Life Sciences, Batavia, IL). VH was filter-sterilized with a 0.45 µm PES sterile filter (Whatman, Clifton, NJ). Sterile AH and VH were stored at −80°C until use. For microarray experiments, aspirates were pooled to a total of 40 mL achieve adequate volume.

### Microarray analysis

*S. aureus* strains were struck from freezer stock onto BHI agar and incubated overnight. Colonies were used to inoculate CDM, AH or VH broth cultures, which were incubated overnight and then used to inoculate new CDM, AH and VH broth cultures to an initial OD_600_ of 0.02–0.03. Bacteria were harvested for microarray analysis and semi-quantitative RT-PCR analyses during exponential growth, at an OD_600_ of 0.4–0.5 for CDM and 0.15–0.2 for AH and VH. Cells were stabilized with RNAProtect (Qiagen) and RNA was extracted using the RNA Bee reagent (TelTest, Inc.) per the manufacturer's instructions. Absence of DNA contamination was verified by PCR using primers targeting the 16S rRNA gene (For, 5′-AAC TCT GTT ATT AGG GAA GAA C-3′; Rev, 5′-CCA CCT TCC TCC GGT TTG TCA CC-3′). cDNA synthesis, fragmentation, biotin labeling and hybridization to Affymetrix *S. aureus* GeneChips were performed as previously described [27]. Hybridization and scanning of GeneChips were performed at the University of Iowa DNA Core. Microarray experiments were performed in duplicate. Affymetrix GeneChip data was analyzed with GeneChip Operating Software (GCOS version 1.4). Probe sets with statistically significant change calls (increased or decreased; p≤0.05) between control and test conditions were considered for further analysis, and fold change cut-offs were applied as described in the text. Microarray data have been deposited in ArrayExpress under accession numbers E-MTAB-2928.

For the Affymetrix *S. aureus* GeneChip, probe set IDs (for example, sa_c10261s8939_a_at) are used instead of gene names or ORF designations. To convert probe set IDs to genomic loci, we downloaded target DNA sequences corresponding to differentially expressed probe sets from the NetAffx Analysis Center (www.affymetrix.com/analysis/index.affx). Target sequences were compared to available *S. aureus* sequences in GenBank using NCBI BLAST (http://blast.ncbi.nlm.nih.gov). Transcription unit and metabolic pathway predictions were obtained from the BioCyc *Staphylococcus aureus* COL database (http://biocyc.org/organism-summary?object=SAUR93062). Where appropriate, predicted protein products of differentially expressed genes were analyzed for putative functions using NCBI protein BLAST and Pfam 26.0 (http://pfam.janelia.org). Subcellular localization of proteins was predicted using PSORTb version 3.0 (http://www.psort.org/).

### Semiquantitative RT-PCR

Semiquantitative reverse transcription (RT)-PCR was performed using Superscript II reverse transcriptase as outlined by the manufacturer (Invitrogen). *S. aureus* RNA was used to make cDNA with priming by random hexamers. cDNA was purified with the QIAquick PCR Purification kit (Qiagen). Five ng of the resulting purified cDNA was used as template in a 25 µl standard PCR reaction with Taq polymerase (New England Biolabs). For visualization, 5 µl of the PCR reaction was analyzed using agarose gel electrophoresis with ethidium bromide. The housekeeping gene *clpX* was used as a control for gene expression. Expression of *tst*, *cidA* and *nanA* was evaluated. Those genes were chosen based on their significant differential regulation across media (*nanA* was 54.8 in AH vs DM; *cidA* was 12.8 in VH vs DM; *tst* was 15.2 in AH vs DM). An independent set of pooled fluids was used for this experiment.

### Murine AC infection model

Female C57BL/6J mice were obtained from the Charles River Laboratory (Boston, MA). All animals were treated according to the guidelines of the Association for Research in Vision and Ophthalmology Resolution on the Use of Animals in Research. The protocol was approved by Massachusetts Eye and Ear Infirmary’s Institutional Animal Care and Use Committee (IACUC). Mice were anesthetized by intraperitoneal injection of ketamine (62.5 mg/kg) and xylazine (12.5 mg/kg). Animals were euthanized at the appropriate time points by CO_2_ asphyxiation.

*S. aureus* colonies obtained after growth on BHI agar were cultured overnight in BHI broth and subcultured 1∶500 with fresh BHI broth and grown to an OD_600 nm_ of 0.4–0.8, pelleted by centrifugation at 10,000 rpm, and washed twice with PBS. The ACs of the right eyes of 6–8-week-old female mice were inoculated with 1 µL of *S. aureus* culture using 35 gauge needle on a nanofil syringe (World Precision Instruments, Inc.), just anterior to the limbus without touching the iris. For *S. aureus* MS7, 2 mM isopropyl β-D-1-thiogalactopyranoside (IPTG) was added to subcultures and included in PBS washes. The left eyes were left untreated and served as internal controls for electroretinography (ERG). Experiments were performed at least in duplicate with a minimum of 3 animals per experimental group. Animals were given water with 12 mM IPTG for one week prior to infection with *S. aureus* MS7. Quantification of in vivo bacterial growth, slitlamp examination and ERG were performed as described previously [28]. Briefly, intraocular inflammation was graded using the following criteria: 0, normal; 1, small amount of fibrin on the pupil; 2, iris partially covered with fibrin and/or hypopyon; 3, iris covered with fibrin and/or hypopyon; 4, pupil not visible. The retinal function in the infected eye was measured using ERG and was defined as the ratio of the b-wave (measured from the trough of the a-wave to the peak of the b-wave) amplitude of the experimentally treated eye to that of the contralateral untreated eye, times 100.

### Histological analysis

Enucleated eyes were fixed in buffered formalin solution and histological analysis was performed by Excalibur Pathology Inc. (Oklahoma City, OK). Pathology slides were examined for signs and extent of inflammation in the different compartments of the eye, and disruption in retinal architecture.

### Statistical analysis

Normality tests were performed on all data sets. The data were analyzed with an unpaired t-test if the distribution was Gaussian, or with the nonparametric Mann-Whitney test if the data were not normally distributed. P≤0.05 was set as the basis for rejection of the null hypothesis. The statistical analysis was conducted with the aid of the Harvard Catalyst Biostatistical Consulting Program.

## Results

### *S. aureus* SA564 *in vitro* growth in bovine AH and VH

**Figure 1:**
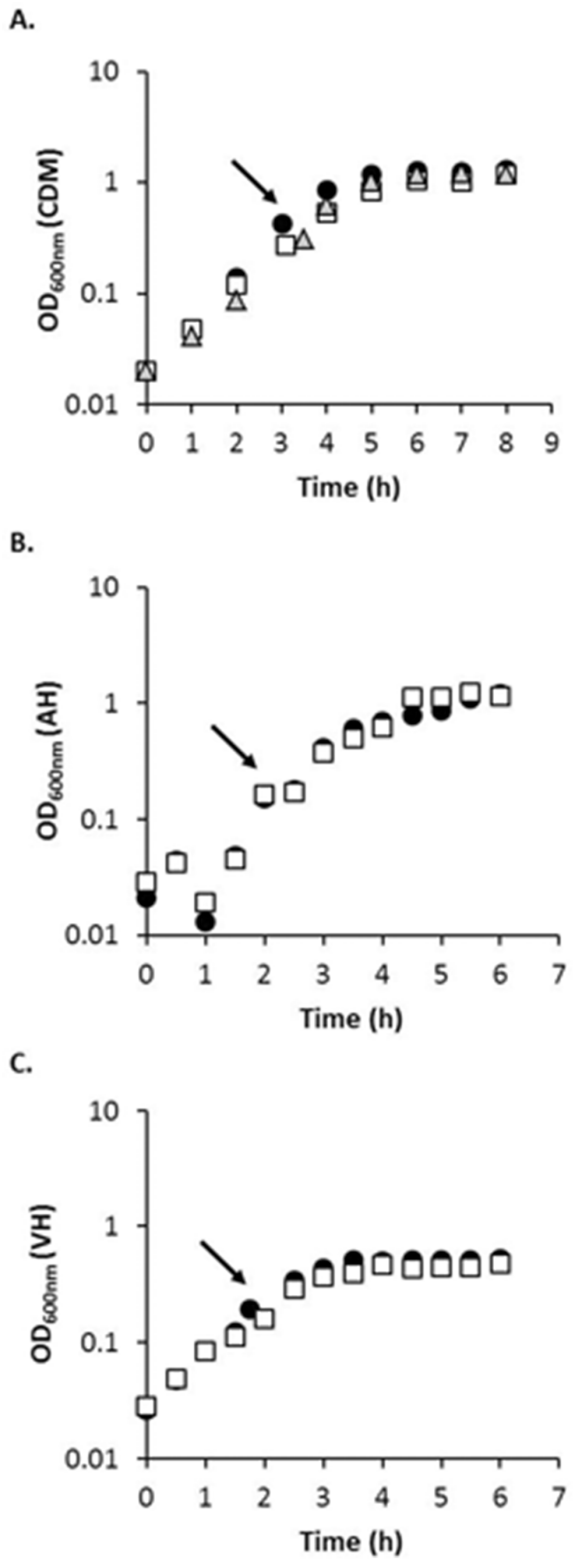
*S. aureus* growth in CDM, AH and VH. *S. aureus* SA564 (black circles), CDM7 (white squares), and MS7 (grey triangles) were grown in CDM, AH, or VH, as described in the text. Growth was monitored by optical density at 600 nm (OD_600 nm_). Representative growth curves are shown. Arrows indicate time points where microarray sampling occurred.

*S. aureus* SA564 is a clinical isolate that was previously used to evaluate a role for *codY* in *S. aureus* virulence regulation [29]. As a first step in understanding *S. aureus* physiology and metabolism during endophthalmitis, we evaluated *S. aureus* SA564 growth in pooled AH and VH harvested from commercially obtained bovine eyes. *S. aureus* SA564 was also grown in Sokransky's medium, a buffered, defined medium supplying amino acids, vitamins, nucleobases, metals, and other growth factors [25], supplemented with 20 mM glucose as a carbon source. For the purposes of this manuscript, Sokransky's medium with 20 mM glucose is referred to as CDM. Representative growth curves for *S. aureus* SA564 as assessed by OD_600 nm_ are shown in Fig. 1. These data demonstrate that CDM, pooled bovine AH, and pooled bovine VH support *in vitro* growth of *S. aureus* SA564. We reproducibly observed a clumping phenotype early in *S. aureus in vitro* AH growth, resulting in reduced cell density measurements obtained by OD_600 nm_, as shown in Fig. 1. Also of note, one pooled AH sample did not support robust *S. aureus in vitro* growth (data not shown), possibly as a result of antimicrobials such as antibiotics or inflammatory factors present in one or more individual AH samples. We were unable to obtain additional information from the vendor about the health and history of cows used in this study.

### Transcriptome analysis of AH- and VH-grown *S. aureus* SA564

We used Affymetrix GeneChips to examine gene expression of *S. aureus* SA564 during growth in bovine AH and VH, using CDM-grown SA564 as a control. Cells were harvested for microarray analysis during exponential growth; representative time points are indicated by arrows in Fig. 1. Microarrays were performed in duplicate for each growth condition. The *S. aureus* GeneChip was designed using genome sequence from the *S. aureus* strains N315, Mu50, NCTC 8325, and COL, and queries over 3300 ORFs and intergenic regions [30]. Genome sequence data are not available for *S. aureus* SA564. For transcriptome experiments with wild-type SA564 and its isogenic *codY* mutant CDM7 (discussed further below), we obtained statistically significant hybridization over the background for an average of 64.8% of probe sets (range, 57–71.8% over 12 chips), corresponding to ∼3763 probe sets queried.

We performed three comparative analyses of wild-type *S. aureus* SA564 transcriptomes: AH-grown cells compared to CDM-grown cells (to model growth of *S. aureus* in the anterior chamber), VH-grown cells compared to CDM-grown cells (to model growth of *S. aureus* in the posterior chamber), and VH-grown cells compared to AH-grown cells (to model transcriptional changes potentially occurring after translocation of *S. aureus* from the anterior to posterior chambers). A fold change cut-off of 10 was used to consider the most highly differentially regulated genes in each condition. For the *S. aureus* GeneChip, probe set IDs (for example, sa_c10261s8939_a_at) are used instead of gene names or ORF assignments. To convert differentially expressed probe set IDs to meaningful *S. aureus* genomic loci, we compared target DNA sequences corresponding to differentially expressed probe sets to *S. aureus* sequences in GenBank (see Materials and Methods).

A total of 78 unique probe sets corresponding to 72 ORFs, regulatory RNAs and intergenic regions were differentially regulated at least 10-fold across the three comparisons (Table 2 and Table S1). Table S1 is an expanded version of Table 2 showing probe set IDs, BLAST hit distribution among *S. aureus* COL, Mu50, N315, and NCTC 8325 genomes, and fold change data for every gene shown in Table 2, irrespective of meeting the fold change cut-off of 10. Fold changes ≥3 and <10 are also shown in Table 2 and are italicized. Two probe sets identified as being differentially regulated in the VH versus CDM analysis, sa_i7808d_x_at and sa_i9119u10r_x_at, query similar sequence at non-syntenic regions of multiple *S. aureus* genomes and could not be assigned to a single genomic locus (Table S1). Eleven of the differentially expressed probe sets identified for the two comparisons using CDM-grown cells as controls had high standard deviations (Table S1). Further investigation of the CDM control arrays revealed that those 11 probe sets were themselves differentially expressed between the two CDM controls (Table S1). Data for those 11 probe sets are shown only in Table S1. No other potential conflicts were detected in the control CDM arrays.

**Table 2:** Transcriptomes of AH- and VH-grown *S. aureus* SA564.

ORF/intergenic region^a^	Gene^a^	Description of gene or queried region	AH vs DM Fold change^b^	VH vs DM Fold change^c^	VH vs AH Fold change^d^
Genes putatively involved in nutrient transport or metabolism					
SACOL0154	*aldA1*	Aldehyde dehydrogenase	13.2 (3.3)	*5.0 (1.1)*	
SACOL0173	*ipdC*	Indole-3-pyruvate decarboxylase	12.1 (2.2)	12.1 (1.3)	
SACOL0176		Conserved hypothetical protein	42.2 (1.1)	*3.9 (1.3)*	−10.9 (1.3)
SACOL0177	*murQ*	Glucokinase regulator-related protein	27.4 (1.2)		−11.5 (1.2)
SACOL0178^e^		PTS system, IIBC components	21.1 (1.2)		−*8.3 (1.3)*
SACOL0179		Phosphosugar-binding transcriptionalregulator, RpiR family	12.1 (1.2)		−*6.2 (1.5)*
SACOL0192		Maltose ABC transporter, ATP-binding protein, putative	21.5 (1.5)		−*6.5 (1.6)*
SACOL0193		Maltose ABC transporter, maltose-bindingprotein, putative	14.4 (1.3)	*3.4 (1.2)*	−*4.2 (1.4)*
SACOL0194		Maltose ABC transporter permease protein	11.1 (1.5)	*3.3 (1.4)*	−*3.7 (1.4)*
SACOL0195		Maltose ABC transporter permease protein	13.9 (1.5)	*3.7 (1.4)*	−*3.4 (1.5)*
SACOL0196		Oxidoreductase, Gfo/Idh/MocA family	12.6 (1.5)	*3.3 (1.3)*	−*3.9 (1.5)*
SACOL0197		Oxidoreductase, Gfo/Idh/MocA family	10.2 (1.4)	*3.2 (1.1)*	−*3.2 (1.4)*
SACOL0198		Conserved hypothetical protein	10.7 (1.4)	*3.1 (1.3)*	−*3.1 (1.2)*
SACOL0200^e^		Phosphoglycerate transporter family protein	44.5 (1.4)		−27.9 (1.6)
SACOL0204	*pflB*	Formate acetyltransferase	16.0 (2.7)		−*4.4 (2.0)*
SACOL0205	*pflA*	Pyruvate formate-lyase-activating enzyme	11.5 (2.5)	*4.3 (1.8)*	
SACOL0215		Propionate CoA-transferase, putative		10.7 (1.4)	
SACOL0308^e^	(*yeiC*)	Carbohydrate kinase, PfkB family	54.8 (1.5)		−40.1 (1.8)
SACOL0309	(*yeiN*)	Conserved hypothetical protein	45.3 (1.6)		−38.7 (1.7)
SACOL0310	(*yeiM*)	Nucleoside permease NupC, putative	28.3 (2.1)		−32.0 (1.7)
SACOL0311	*nanT*	Sodium:solute symporter family protein	38.1 (1.7)		−13.2 (1.7)
SACOL0312	*nanA*	N-acetylneuraminate lyase	54.8 (1.7)	*3.5 (1.3)*	−14.4 (1.7)
SACOL0400	(*ulaA*)	Transport protein SgaT, putative	13.7 (1.4)		−12.6 (1.4)
SACOL0401	(*ulaB*)	Conserved hypothetical protein	20.7 (1.4)		−16.0 (1.5)
SACOL0402	(*ulaC*)	PTS system, IIA component	29.3 (1.4)		−23.8 (1.4)
SACOL0403		Transcriptional antiterminator, BglGfamily	28.3 (1.4)		−28.3 (1.6)
ig_SACOL0913		Intergenic region downstream ofSACOL0913	−15.2 (1.9)	−*6.3 (2.4)*	
SACOL0960	*rocD2*	Ornithine aminotransferase	10.0 (2.0)	*3.5 (1.2)*	
SACOL1360		Aspartate kinase	10.6 (5.9)	*6.0 (1.3)*	
SACOL1734	*gapA2*	Glyceraldehyde-3-phosphate dehydrogenase	10.4 (4.9)		−*6.8 (4.9)*
SACOL1784	*acuA*	Acetoin utilization protein AcuA	13.7 (3.6)		
SACOL1785		Acetoin utilization protein AcuC	10.6 (3.2)		
SACOL1816	*putA*	Proline dehydrogenase	21.1 (1.7)	*3.7 (1.4)*	−*5.4 (1.7)*
SACOL2163		Conserved hypothetical protein	11.7 (1.4)	*7.2 (1.5)*	
SACOL2247		Hypothetical protein	−19.7 (7)		
SACOL2356		ABC transporter, ATP-binding protein			−10.0 (2.7)
SACOL2357		ABC transporter, permease protein			−10.6 (2.9)
SACOL2403		ABC transporter, substrate bindingprotein	−*6.7 (1.5)*	−15.7 (1.5)	
SACOL2415	*gpm*	Phosphoglycerate mutase	10.9 (1.7)	12.1 (1.6)	
SACOL2427	*bioA*	Adenosylmethionine-8-amino-7-oxononanoate aminotransferase	*8.3 (3.5)*	13.9 (1.5)	
SACOL2428	*bioD*	Dethiobiotin synthase	16.0 (3.9)	24.3 (1.7)	
SACOL2441		Amino acid permease	10.6 (4.4)	*3.9 (1.7)*	
SACOL2527		Fructose-1,6-bisphosphatase, putative	11.3 (2)	*6.7 (1.2)*	
Putative or confirmed virulence and biofilm factors					
SACOL0247	*lrgA*	Holin-like protein LrgA	*7.0 (3.8)*		−14.4 (4.0)
SACOL0248	*lrgB*	LrgB protein	*6.3 (4.1)*		−11.1 (3.8)
SACOL1187		Antibacterial protein (phenol soluble modulin)	10.4 (2.5)		−*6.5 (1.8)*
SACOL2509	*fnbB*	Fibronectin binding protein B		−13.0 (1.3)	
SACOL2554_1	*cidA*	LrgA family protein		12.8 (1.4)	34.9 (1.4)
SACOL2652	*clfB* (rev comp)	Queries 97 nt region complementaryto the 5′ end of *clfB* (clumping factor B)	−30.4 (1.2)	−*6.4 (2.0)*	
SACOL2694	*geh*	Lipase	19.4 (1.4)	*3.7 (1.3)*	−*4.4 (1.5)*
SA1817^f^	*sec3*	Enterotoxin type C3	10.9 (3.0)		−*6.9 (2.4)*
SA1819^f^	*tst*	Toxic shock syndrome toxin-1	15.2 (5.5)		−*8.3 (5.7)*
Probable prophage or genomic island genes of unknown significance					
SACOL0325^g^		Prophage L54a, antirepressor, putative	−23.8 (2.2)	−*9.9 (2.0)*	
SACOL0326^g^		Hypothetical protein	−26.4 (2.4)	−15.7 (2.2)	
SAOUHSC_02028 e		φETA ORF57-like protein	−22.6 (1.9)	−25.5 (1.4)	
SAOUHSC_02078		φPV83 orf 10-like protein	−20.0 (2.0)	−10.4 (1.8)	
SAOUHSC_02206		Hypothetical phage protein	−10.4 (1.6)	−*9.5 (1.6)*	
SAV0859^h^		Hypothetical protein	−17.1 (1.7)	−*8.0 (2.3)*	
ig_SAV0860^h^		Intergenic region downstream of SAV0860	−14.2 (1.8)	−10.7 (1.9)	
SAV0905^h^		Similar to φETA ORF57-like protein	−22.6 (1.9)	−25.5 (1.4)	
SAV1985^i^		Hypothetical protein	−17.4 (1.9)	−11.5 (2.0)	

a ORFs were identified by BLAST analysis of Affymetrix array target sequences, as described in the materials and methods. If a corresponding ORF was identified in *S. aureus* COL, that strain's ORF identifiers were used as default. SACOL####, *S. aureus* COL (GenBank accession number CP000046.1); SAV####, *S. aureus* Mu50 (BA000017.4); SA####, *S. aureus* N315 (BA000018.3); SAOUHSC_####, *S. aureus* NCTC 8325 (CP000253.1). Vertical lines indicate genes computationally predicted to be in the same transcriptional unit. Gene names in brackets were assigned in this study using data shown in Table S1.

b Fold change in expression of genes during *S. aureus* SA564 growth in AH as compared to growth in glucose-supplemented CDM; a positive number indicates an up-regulation of the gene during growth in AH. Standard deviation is shown in parentheses. Fold changes ≥3 and <10 are shown and italicized.

c Fold change in expression of genes during *S. aureus* SA564 growth in VH as compared to growth in glucose-supplemented CDM; a positive number indicates an up-regulation of the gene during growth in VH. Standard deviation is shown in parentheses. Fold changes ≥3 and <10 are shown and italicized.

d Fold change in expression of genes during *S. aureus* SA564 growth in VH as compared to growth in AH; a positive number indicates an up-regulation of the gene during growth in VH. Standard deviation is shown in parentheses. Fold changes ≥3 and <10 are shown and italicized.

e At least two differentially expressed probe sets were assigned to these ORFs. Data for all probe sets are shown in Table S1.

f On genomic island *v*SA4 [48].

g On φCOL [48].

h On φSa1 [48].

i On φSa3 [48].

The differentially expressed genes identified by our microarray analysis can be divided into three categories: (1) genes putatively involved in transport or metabolism of nutrients; (2) putative or confirmed virulence and/or biofilm factors; and (3) probable prophage or genomic island genes of unknown significance. Perhaps not surprisingly, most differentially expressed genes in the analysis were assigned to the first category.

Several putative transcriptional units are highly up-regulated during growth in AH as compared to CDM, and are also down-regulated during VH growth compared to AH, suggesting that the corresponding carbon substrates may be specific to or more abundant in AH. These include SACOL0308-0310, putatively involved in pseudouridine transport and catabolism; SACOL0311-0312, encoding an operon required for sialic acid catabolism in *S. aureus* [30]; and SACOL0400-0403, putatively involved in ascorbate uptake. In addition to these genes, SACOL0176-0179, SACOL0192-0195, and SACOL0200 are upregulated during growth in AH, and each appear to involved in the uptake of and transcriptional response to sugars or phosphosugars (Table S1).

We additionally identified genes putatively involved in lysine biosynthesis from aspartate (SACOL1360, *bioA*, *bioD*) and gluconeogenesis (*gpm*, SACOL2527) as being up-regulated during growth in both AH and VH as compared to CDM, suggesting that their regulation is specific to growth in ocular fluids. Another gene putatively involved in gluconeogenesis, *gapA2*, was also differentially regulated, but its up-regulation was specific to AH growth.

We also observed differential regulation of a set of putative prophage and/or genomic island genes (Table 2). Interestingly, all of these genes were down-regulated in both AH and VH relative to growth in CDM, suggesting that increased expression of these genes is CDM-specific. The significance of this is unknown. Additionally, because genome sequence is not available for SA564 and thus the extent of its accessory genome is unknown, we cannot exclude the possibility of AH- and/or VH-specific differential regulation of prophage, plasmid and island genes that are not represented on the *S. aureus* Affymetrix chip.

**Figure 2:**
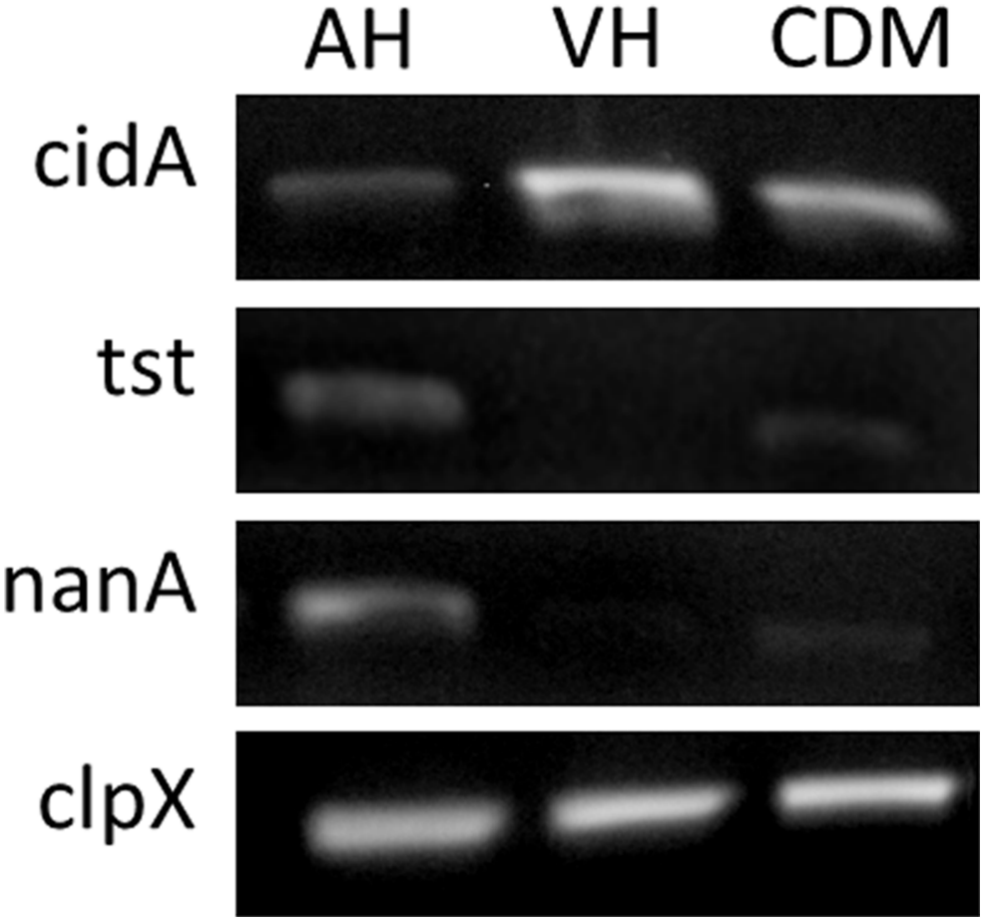
Semi-quantitative RT-PCR. Differential expression of *tst*, *nanA* and *cidA* in AH, VH and CDM. *clpX* was used as a constitutively expressed control gene.

Our microarray analysis was verified using semiquantitative RT-PCR to verify expression of a few genes discussed including *tst*, *cidA* and *nanA* (Figure 2). A more intense signal was observed for *tst* and *nanA* from AH samples when compared to CDM, and *cidA* signal was more intense in VH when compared to CDM. The results are consistent with the differential regulation of those genes in our microarray analysis.

### The *S. aureus* SA564 *codY* mutant in CDM, AH, and VH

We speculated that *codY* might have a role in ocular infections given the presence of leucine, isoleucine and valine in human AH and VH [16], [17], [31], [32] and given the possibility that those substrates might become depleted during *in vivo S. aureus* growth. We first explored the effect of *codY* deletion on SA564 transcriptional responses to CDM, bovine AH and bovine VH, using the previously described SA564 *codY*-mutant strain, CDM7 [29]. Using GeneChip analysis, we identified 130 probe sets as being differentially expressed ≥5-fold, corresponding to 125 ORFs, regulatory RNAs and intergenic regions (Table 3 and Table S2).

**Table 3:** Genes differentially expressed by the *S. aureus* SA564 *codY* mutant during growth in CDM, AH and VH.

ORF^a^	Gene	Description of gene or queried region	DM Fold change^b^	AH Fold change^b^	VH Fold change^b^
SA1817	*sec3*	Enterotoxin type C3	6.7 (1.2)		
SACOL0136	*cap5A*	Capsular polysaccharide biosynthesis protein Cap5A	8.0 (1.6)		
SACOL0138	*cap5C*	Capsular polysaccharide biosynthesis protein Cap5C	6.2 (1.9)		
SACOL0185		Peptide ABC transporter permease	53.8 (1.3)	6.5 (1.9)	11.5 (1.2)
SACOL0186		Peptide ABC transporter permease	31.5 (1.2)	5.9 (1.9)	10.9 (1.1)
SACOL0187		RGD-containing lipoprotein	29.3 (1.3)		11.9 (1.1)
SACOL0188	*ggt*	γ-glutamyltranspeptidase	13.0 (1.2)		7.1 (1.1)
SACOL0214		Long-chain-fatty-acid-CoA ligase			6.3 (2.4)
SACOL0215		Propionate CoA-transferase			8.4 (1.5)
SACOL0222	*ldh1*	L-lactate dehydrogenase	7.9 (2.2)		
SACOL0267		Hypothetical protein	7.2 (1.0)		
SACOL0270		Staphyloxanthin biosynthesis protein	6.5 (1.4)		
SACOL0271		Hypothetical protein	5.3 (1.1)		
SACOL0274		Hypothetical protein	5.0 (3.2)		
SACOL0308^c^		Carbohydrate kinase (3′ region)		−7.2 (1.7)	
SACOL0309		Hypothetical protein		−6.8 (1.7)	
SACOL0310		Nucleoside permease NupC		−7.2 (2.0)	
SACOL0427		Hypothetical protein	7.5 (1.1)		
SACOL0428	*metE*	5-methyltetrahydropteroyltriglutamate/homocysteineS-methyltransferase	19.7 (1.2)		9.2 (1.6)
SACOL0429		Bifunctional homocysteineS-methyltransferase/5,10-methylenetetrahydrofolate reductase	28.3 (1.2)		11.5 (1.4)
SACOL0430		Trans-sulfuration enzyme family protein	36.1 (1.1)		12.8 (1.1)
SACOL0431		Trans-sulfuration enzyme family protein	30.9 (1.1)		10.4 (1.1)
SACOL0431 rev comp		Reverse complement of interior105 nt region of SACOL0431	11.1 (1.4)		
ig_SACOL0431-2		121 nt region upstream of SACOL0431	12.3 (1.7)		
SACOL0480		Hypothetical protein	9.7 (1.2)		
SACOL0502		Cysteine synthase/cystathionine beta-synthase	7.6 (2.0)		
SACOL0503^c^	*metB*	Cystathionine γ-synthase	7.1 (2.0)		
SACOL0504		ABC transporter ATP-binding protein	30.4 (2.1)		
SACOL0505		ABC transporter permease	29.3 (2.4)		
SACOL0506		ABC transporter substrate-binding protein	18.4 (1.5)		
SACOL0514	*gltB*	Glutamate synthase	23.4 (1.3)		12.1 (1.5)
SACOL0515	*gltD*	Glutamate synthase, small subunit	15.7 (1.3)		9.8 (1.2)
ig_SACOL0701-2 rev comp		Reverse complement of intergenicregion between SACOL0701-2; 5′106 nt overlap RsaD sRNA fromGeissmann, et al. 2009 Nucleic Acids Res	20.4 (2.1)	5.4 (1.4)	9.2 (1.4)
SACOL0796		Iron compound ABC transporter permease	−5.5 (2.3)		
SACOL0797		Iron compound ABC transporter permease	−5.8 (2.0)		
SACOL0798		Iron compound ABC transporter ATP-binding protein	−6.0 (2.3)		
SACOL0815		Ribosomal subunit interface protein	5.5 (1.7)		
SACOL0860	*nuc*	Thermonuclease precursor	9.2 (1.2)		5.3 (1.3)
SACOL0991	*oppB*	Oligopeptide ABC transport permease	10.7 (1.3)		
SACOL0992	*oppC*	Oligopeptide ABC transporter permease	11.5 (1.4)		
SACOL0993	*oppD*	Oligopeptide ABC transporter ATP-binding protein	8.3 (1.3)		
SACOL0994	*oppF*	Oligopeptide ABC transporter ATP-binding protein	7.9 (1.2)		
SACOL0995		Oligopeptide ABC transporter oligopeptide-binding protein	8.3 (1.2)		
SACOL1018		Sodium:alanine symporter family protein	21.9 (1.2)	9.9 (3.4)	15.7 (1.7)
ig_SACOL1018-9		Intergenic region betweenSACOL1018 and SACOL1019		12.3 (1.7)	21.9 (3.7)
SACOL1019		Hypothetical protein	6.1 (1.1)		
SACOL1033		Hypothetical protein	8.9 (1.5)		
SACOL1038		Membrane protein	9.5 (1.2)	6.3 (2.1)	12.3 (1.3)
SACOL1039		Hypothetical protein	7.0 (1.2)	5.6 (1.6)	13.0 (1.5)
SACOL1040		ABC transporter ATP-binding protein	9.4 (1.5)	7.6 (2.0)	8.4 (1.6)
SACOL1186		Antibacterial protein (phenol soluble modulin)	6.8 (2.6)		
SACOL1187		Antibacterial protein (phenol soluble modulin)	6.8 (2.4)		
SACOL1272^c^	*codY*	Transcriptional repressor CodY	−789.6 (1.1)	−652.6 (1.4)	−197.4 (1.5)
SACOL1360		Aspartate kinase	30.4 (1.3)		
SACOL1362	*hom*	Homoserine dehydrogenase	16.3 (1.3)		
SACOL1363	*thrC*	Threonine synthase	17.5 (1.4)		
SACOL1364	*thrB*	Homoserine kinase	14.9 (1.3)		
SACOL1368	*katA*	Catalase	5.3 (1.7)		
SACOL1403	*trpE*	Anthranilate synthase component I	6.0 (1.4)		
SACOL1404	*trpG*	Anthranilate synthase component II	6.4 (1.5)		
SACOL1405	*trpD*	Anthranilate phosphoribosyltransferase	8.6 (1.6)		6.5 (1.1)
SACOL1406	*trpC*	Indole-3-glycerol-phosphage synthase	13.7 (1.5)		7.7 (1.2)
SACOL1407	*trpF*	N-(5′-phosphoribosyl)anthranilate isomerase	21.9 (1.5)		10.0 (1.3)
SACOL1408	*trpB*	Tryptophan synthase subunit β	18.4 (1.7)		6.7 (1.2)
SACOL1409	*trpA*	tryptophan synthase subunit α	6.1 (1.4)		
SACOL1428	*lysC*	Aspartate kinase	10.9 (1.7)		
SACOL1429	*asd*	Aspartate semialdehyde dehydrogenase	14.2 (1.4)		
SACOL1430	*dapA*	Dihydrodipicolinate synthase	13.5 (1.2)		
SACOL1431	*dapB*	Dihydrodipicolinate reductase	14.4 (1.2)		
SACOL1432	*dapD*	2,3,4,5-tetrahydropyridine-2,6-dicarboxylateN-succinyltransferase	13.0 (1.1)		
ig_SACOL1432-3		Intergenic region between *dapD* andSACOL1433	26.4 (1.8)		
SACOL1433		M20/M25/M40 family peptidase	9.2 (1.3)		
SACOL1434		Alanine racemase	9.2 (1.2)		
SACOL1449	*sucA*	2-oxoglutarate dehydrogenase E1 component	5.2 (1.1)		
SACOL1772		Class V aminotransferase	24.3 (1.2)	5.4 (2.9)	13.7 (1.2)
SACOL1773	*serA*	D-3-phosphoglycerate dehydrogenase	21.5 (1.3)		11.9 (1.2)
SACOL2003	*hlb*	Queries 170 nt (positions 19–188) in5′ region of *hlb* (phospholipase C)	5.6 (1.3)		
SACOL2021-2	RNAIII	3′ 345 nt of RNAIII; probes regiondownstream of δ-hemolysin gene	12.1 (3.2)		
SACOL2022	*hld*	δ-hemolysin	10.4 (3.8)		
SACOL2031		Ammonium transporter family protein	11.1 (1.3)	5.0 (1.9)	5.6 (1.2)
ig_SACOL2041-2		Intergenic region upstream ofSACOL2042 *ilvD* (*ilvD* promoterregion)	23.4 (4.3)		
SACOL2042	*ilvD*	Dihydroxy-acid dehydratase	48.5 (1.4)	11.3 (5.4)	26.4 (1.4)
SACOL2043	*ilvB*	Acetolactate synthase large subunit	80.2 (1.2)		39.4 (1.2)
SACOL2044		Acetolactate synthase 1 regulatory subunit	157.6 (1.7)	8.6 (3.1)	85.9 (1.4)
ig_SACOL2044-5		Intergenic region between SACOL2044 and *ilvC*			33.7 (1.3)
SACOL2045	*ilvC*	Ketol-acid reductoisomerase	100.4 (1.3)	5.3 (2.2)	61.8 (1.3)
SACOL2046	*leuA*	2-isopropylmalate synthase	109.5 (1.2)		64.0 (1.3)
SACOL2047	*leuB*	3-isopropylmalate dehydrogenase	89.0 (1.3)	5.2 (2.3)	64.0 (1.4)
SACOL2048	*leuC*	Isopropylmalate isomerase large subunit	95.3 (1.2)		62.9 (1.4)
SACOL2049	*leuD*	Isopropylmalate isomerase small subunit	107.6 (1.2)	5.2 (1.7)	76.1 (1.2)
SACOL2050	*ilvA2*	Threonine dehydratase	78.8 (1.2)	5.2 (1.9)	41.5 (1.2)
SACOL2314		Sodium/bile acid symporter family protein	7.7 (1.1)		
SACOL2403		ABC transporter substrate-binding protein	−5.5 (1.4)		
SACOL2554.1		LrgA family protein			−5.0 (1.5)
SACOL2585		Hypothetical protein	20.0 (1.2)	7.7 (2.8)	10.2 (1.5)
ig_SACOL2585-4		Intergenic region downstream of SACOL2585	6.1 (1.1)		
SACOL2619		Amino acid permease	19.0 (1.2)	6.6 (2.9)	9.2 (1.7)
SACOL2620		4-aminobutyrate aminotransferase	29.3 (1.3)	9.0 (3.6)	16.0 (1.5)
ig_SACOL2620-1		Intergenic region upstream of SACOL2620	34.3 (1.3)	7.9 (3.4)	20.4 (2.1)
SACOL2627	*betA*	Choline dehydrogenase	5.0 (2.4)		
SACOL2628	*betB*	Betaine aldehyde dehydrogenase	5.4 (2.4)		
SACOL2641	*gpxA2*	Glutathione peroxidase	6.4 (1.4)		
ig_SACOL2641-2		Intergenic region betweenSACOL2642 and *gpxA2*(SACOL2641)	8.3 (1.6)		
ig_SACOL2641-2 rev comp		Reverse complement of intergenicregion between SACOL2642 and*gpxA2*	6.5 (1.8)		
SACOL2659	*aur*	Zinc metalloproteinase aureolysin	12.3 (1.6)	5.1 (1.4)	9.2 (1.5)
SACOL2689	*icaA*	N-glycosyltransferase	6.7 (1.1)		
SACOL2690	*icaD*	Intercellular adhesion protein D	7.5 (1.3)		
ig_SACOL2695-6		Intergenic region betweenSACOL2696 (*hisI*) and SACOL2695	18.7 (1.5)		6.0 (1.5)
SACOL2696	*hisI*	Bifunctional phosphoribosyl-AMPcyclohydrolase/phosphoribosyl-ATP	14.7 (1.2)		11.5 (1.3)
SACOL2697	*hisF*	Imidazole glycerol phosphatesynthase subunit HisF	16.6 (1.2)		12.3 (1.2)
SACOL2698	*hisA*	1-(5-phosphoribosyl)-5-((5-phosphoribosylamino)methylideneamino) imidazole-4-carboxamide isomerase	16.9 (1.2)		18.4 (1.4)
SACOL2699	*hisH*	Imidazole glycerol phosphate synthase subunit HisH	20.7 (1.1)		14.9 (1.5)
SACOL2700	*hisB*	Imidazoleglycerol-phosphate dehydratase	22.2 (1.0)		13.9 (1.2)
SACOL2701		Histidinol-phosphate aminotransferase	24.2 (1.1)		16.9 (1.2)
SACOL2702	*hisD*	Histidinol dehydrogenase	24.3 (1.1)		14.9 (1.1)
SACOL2703	*hisG*	ATP phosphoribosyltransferase catalytic subunit	27.9 (1.1)		11.5 (1.2)
SACOL2704	*hisZ*	ATP phosphoribosyltransferase regulatory subunit	23.0 (1.1)		16.9 (1.1)
ig_SACOL2704-5		Intergenic region between SACOL2705-4	11.7 (1.9)		8.7 (1.5)
SACOL2705		Hypothetical protein	13.9 (1.3)		10.9 (1.1)
ig_SACOL2705-6		Intergenic region between SACOL2706-5		6.1 (1.8)	
SACOL2706		Hypothetical protein	18.7 (1.1)		19.4 (1.5)
SACOL2707		Cobalt transport family protein	20.0 (1.2)		16.9 (1.3)
SACOL2708		ABC transporter ATP-binding protein	30.4 (1.3)		19.7 (1.1)
SACOL2709		Hypothetical protein	31.5 (1.5)		13.5 (1.3)
SACOL2710		Hypothetical protein	59.7 (1.3)	8.6 (3.6)	24.7 (1.3)

a ORFs were identified by BLAST analysis of Affymetrix array target sequences, as described in the materials and methods. If a corresponding ORF was identified in *S. aureus* COL, that strain's ORF identifiers were used as default. SACOL####, *S. aureus* COL (GenBank accession number CP000046.1); SAV####, *S. aureus* Mu50 (BA000017.4); SA####, *S. aureus* N315 (BA000018.3); SAOUHSC_####, *S. aureus* NCTC 8325 (CP000253.1).

b Fold change in expression of genes by *S. aureus* CDM7 as compared to the wild-type strain during growth in the indicated medium; a positive number indicates an up-regulation of the gene by the *codY* mutant. Standard deviation is shown in parentheses.

c At least two differentially expressed probe sets were assigned to these ORFs. Data for all probes sets are shown in Table S2.

Genes differentially regulated by the *S. aureus* SA564 *codY* mutant as compared to the wild-type strain during exponential growth in CDM are similar to those previously identified for *S. aureus* Newman (1) and *S. aureus* UAMS-1 (2) *codY* mutants during exponential growth in a chemically defined medium and tryptic soy broth, respectively (Table S2). As for Newman and UAMS-1, *codY* inactivation in SA564 results in an up-regulation of amino acid metabolic and virulence genes including BCAA metabolism (*ilvDBC, leuABCD, ilvA*), hemolysins *(hlb*, *hld*), and phenol-soluble modulins (SACOL1186, SACOL1187). As expected based on previous studies on CodY regulation in SA564 [29], we observed up-regulation of *icaA*, RNAIII and *hld*. We also identified expression of an enterotoxin, capsular polysaccharide biosynthesis proteins, metalloproteinase aureolysin and others as being affected by *codY* deletion. All other genes are shown in Table 3. Interestingly, of 117 ORFs, regulatory RNAs and intergenic regions identified as being differentially regulated in CDM when comparing *S. aureus codY*-mutant to SA564, all but one (SA1817, the enterotoxin gene) are core to the four *S. aureus* strains used to generate the GeneChip (Table S2) [33]. However, it is possible that as yet unknown *S. aureus* SA564-specific genes are under CodY control.

Of the 117 ORFs, regulatory RNAs and intergenic regions identified as being differentially regulated in the SA564 *codY* mutant relative to the wild-type strain during growth in CDM, 55 of those were also identified as being differentially regulated during growth in VH (Table 3 and Table S2). Of the 117, only 23 were identified as being differentially regulated during growth in AH. We were curious as to why this expression pattern was observed in AH, and whether it could be explained by a relief of CodY repression occurring during growth in AH at the cell density chosen for our microarray experiments. To explore this further, we returned to the microarray analysis of the SA564 wild-type strain grown in AH, as compared to CDM. Expression data for all differentially expressed genes for the SA564 *codY* mutant during growth in CDM were extracted from each of the four SA564 AH versus CDM analyses (Table S2). Analysis of these data revealed that genes previously identified as being under CodY control [1], [2], [5], [29] were de-repressed in one AH sample (AH2), but not the other (AH1), relative to CDM (Table S2), most likely as a result of BCAA becoming depleted from the pooled AH2 sample. Speculatively, these data suggest that, *in vivo* in the anterior chamber, where BCAA are present [16], [31], CodY repression may limit virulence of *S. aureus* during early stages of infection when cell densities are likely to be low. Future studies that track BCAA concentrations and expression of CodY-regulated genes in *ex vivo* AH samples over the course of *S. aureus* growth could be used to explore this further.

We additionally observed AH-specific up-regulation of *lrgAB* and VH-specific up-regulation of *cidA*. *lrgAB* and *cidA* are involved in coordination of cell death and autolysis, in addition to their role in biofilm development through the release of genomic DNA that becomes a structural component of the biofilm matrix. Note that *lrgAB* expression varied between the two AH gene expression experiments, with low albeit significant up-regulation observed during growth in one AH sample (1.4–2.6 fold up-regulated compared to CDM controls), and comparatively higher up-regulation during growth in another AH sample (17.1–27.8-fold up-regulated compared to CDM controls).

### *codY* deletion enhances *S. aureus* virulence in a murine AC infection model

To assess a potential role for CodY in endophthalmitis, we began by examining the potential for intraocular growth of *S. aureus* after injection into the murine AC. Approximately 5×10^3^ CFU of *S. aureus* SA564 or *codY*-mutant were injected into the murine AC, and bacterial growth was assessed after 24 h by extraction and homogenization of the entire eye (Table 4). In all cases, viable *S. aureus* were recovered (SA564 range, 4×10^2^–2.3–10^6^ CFU; *codY* mutant range, 1×10^2^–1.3×10^6^ CFU). Average *in vivo* growth yields of SA564 and CDM7 were similar (4.6×10^5^ CFU for SA564; 2×10^5^ CFU for CDM7). Thus, after introduction into the anterior chamber, the murine eye was a permissive environment for the survival of each strain. Because the entire eye was homogenized for these experiments, it is unknown whether growth occurred in the anterior chamber, the posterior chamber, or both.

**Table 4:** *S. aureus* SA564, CDM7 and MS7 *in vivo* growth yields.

SA564		CDM7		MS7	
Inoculum (CFU)	24 h (CFU)^a^	Inoculum (CFU)	24 h (CFU)^a^	Inoculum (CFU)	24 h (CFU)^a^
5.5×10^3^	4.0×10^2^	8.3×10^3^	1.0×10^2^	1.2×10^4^	ND^b^
	7.0×10^3^		9.0×10^2^		1.0×10^2^
	2.0×10^4^		1.1×10^3^		1.0×10^2^
	2.6×10^4^		1.2×10^4^		3.0×10^2^
	3.7×10^5^		1.9×10^5^		2.3×10^3^
	5.5×10^5^		6.0×10^5^		2.4×10^5^
	2.3×10^6^		1.3×10^6^		
6.0×10^3^	1.0×10^3^	5.0×10^3^	1.0×10^2^	3.0×10^3^	ND
	3.9×10^3^		2.7×10^3^		ND
	1.3×10^6^		7.8×10^3^		2.0×10^2^
			7.3×10^4^		3.0×10^2^
					6.0×10^2^
					1.2×10^3^
					3.1×10^3^

a Number of CFU recovered per homogenized eye is shown. Each entry represents one eye.

b ND, Not detected. The limit of detection for these experiments was 1×10^2^ CFU.

**Figure 3:**
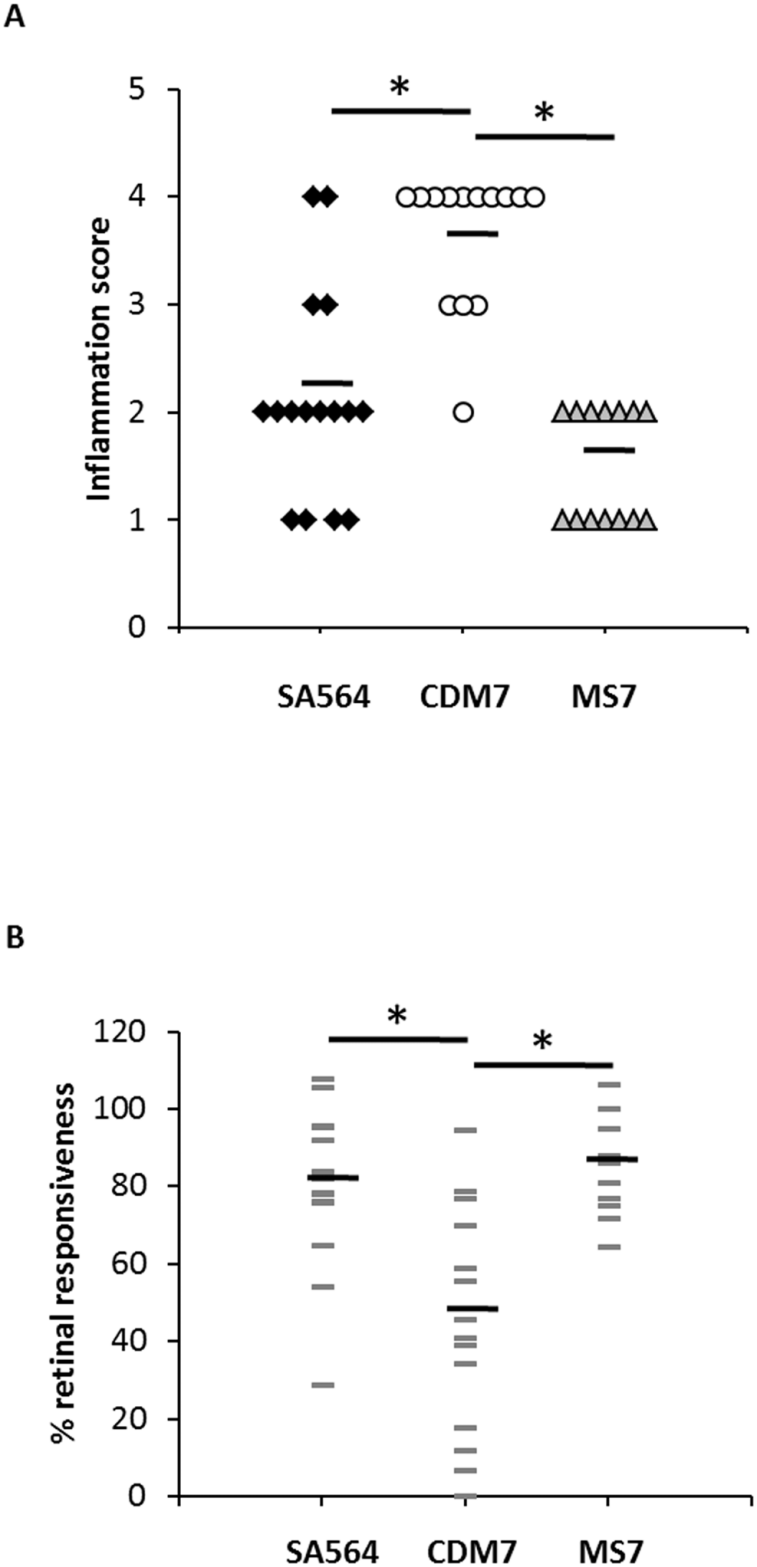
Inflammation and retinal responsiveness in *S. aureus* infected eyes. Inflammation scores (A) and % retinal responsiveness (B) for murine eyes infected with SA564, CDM7, or MS7, assessed 24 h post-inoculation. Average values are indicated by heavy black horizontal lines. *, p≤0.001, Mann-Whitney test.

**Figure 4:**
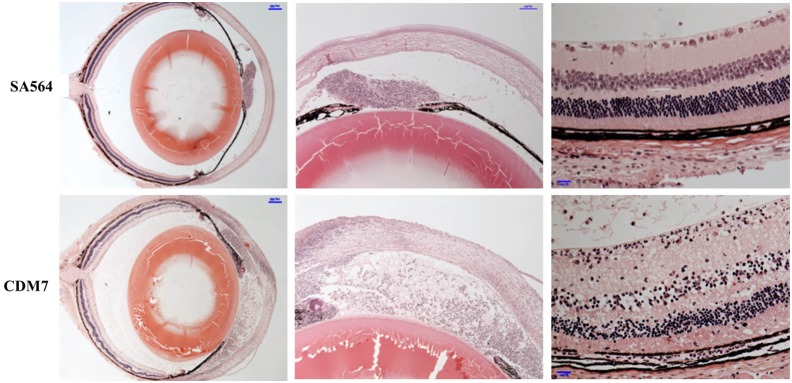
Histology images. Representative histology images of *S. aureus* SA564- and CDM7-infected eyes at 24 h post-inoculation. Retinal responsiveness values for the infected eyes shown were 95.9% (SA564) and 11.9% (CDM7). Panels shown, from top to bottom, are the whole eye, the AC, and the retina.

We next assessed the impact of *codY* on inflammation (Fig. 3A) and retinal responsiveness (Fig. 3B) at 24 h after infection. The distribution of inflammation scores were significantly different for eyes infected with the two strains (*p*<0.001; one-tailed Wilcoxon rank-sum test), with a higher average inflammation score for the *codY* mutant (3.6 versus 2.1) (Fig. 3A). Average retinal responsiveness was lower for eyes inoculated with the *codY* mutant (45% of control versus 80% of control), and the distribution of percent retinal responsiveness values was significantly different between eyes infected with the *codY* mutant and those infected with SA564 (*p* = 0.001, one-tailed Wilcoxon rank-sum test) (Fig. 3B). Representative histology images are shown in Fig. 4. As seen in the figure, the eye infected with the *codY* mutant shows more inflammation in the cornea, anterior chamber, and vitreous, as well as disruption of retinal architecture. Collectively, these data suggest that CodY regulation of its target genes limits *S. aureus* disease in the murine anterior chamber infection model.

Similar experiments were performed using previously described MS7, *codY* complemented strain [29]. In this vector, *codY* expression is under the control of a leaky IPTG-inducible promoter (P_SPAC_) [29]. *codY* expression from pTL6936 appears to be leaky, as partial complementation was observed for an *S. aureus* UAMS-1 *codY* mutant in the absence of IPTG [34]. We observed increased retinal responsiveness and decreased inflammation as assessed by slitlamp for murine eyes inoculated with 10^3^–10^4^ CFU MS7, compared to SA564 and CDM7 (Fig. 4A–B), suggesting that *in vivo* complementation of the *codY* occurred. However, decreased *in vivo* growth yields were observed for MS7 compared to SA564 and CDM7 (Table 4; range, <100–2.5×10^5^ CFU; average, 1.9×10^4^ CFU). Thus it is unknown whether the increased retinal responsiveness and decreased inflammation observed for MS7 were due to complementation of the *codY* lesion or to an *in vivo* growth defect of this strain. MS7 does not have an *in vitro* growth defect relative to CDM7 as assessed by growth in CDM; the average doubling time of MS7 is 49.0±0.5 min, compared to 50.7±0.3 min for CDM7 (Fig. 1).

## Discussion

CodY controls expression of virulence and metabolic genes in response to branched-chain amino acids (BCAA) and GTP. This makes it an important regulatory link between nutrient availability and virulence factor production [1]. However, little is known about its contribution to virulence *in vitro* and *in vivo* in the ocular milieu.

AH is a complex mixture of electrolytes, organic solutes, growth factors, cytokines, and proteins including BCAA that provide the metabolic requirements to the avascular tissues of the anterior segment. It is produced from the non-pigmented ciliary body epithelium through simple diffusion as well as active transport of ions and solutes and exits the anterior chamber mainly via the trabecular meshwork. The volume of human AH in the anterior chamber generally turns over once every 100 minutes replenishing nutrients that have been taken up by avascular ocular tissues and carries away metabolic wastes [16], [17], [31].

In this study, we demonstrated that bovine AH and VH provide adequate nutrition for growth of *S. aureus* SA564, and result in differential gene expression when compared to each other, and to a defined medium. While our *in vitro* bovine AH and VH models lack the nutritional replenishment and immune response that would be characteristic of *in vivo* growth environments, the models are useful in that they allow for the identification of nutrients that *S. aureus* specifically detects and responds to in the ocular milieu, in particular, sialic acid, ascorbate, and pseudouridine.

Pseudouridine is a nucleoside present in RNAs of humans and other animals [32]. It has been detected in tRNAs of the bovine lens [34]. SACOL0308, SACOL0309, and SACOL0310 share protein sequence homology and conserved protein domains with the YeiC, YeiN, and YeiM proteins, respectively, of *Escherichia coli* (Table S1) [35]. YeiC is a pseudouridine kinase, and YeiN is a pseudouridine-5′-phosphate glycosidase. Together, YeiC and YeiN comprise a pathway for the catabolism of pseudouridine to uracil and ribose-5-phosphate [35]. YeiM is a predicted nucleoside transporter and may be involved in uptake of pseudouridine from the environment. It is possible that *S. aureus* SA564 catabolizes pseudouridine for energy (via ribose-5-phosphate), and/or for uracil scavenging in AH.

N-acetylneuraminate, another highly upregulated gene in AH, is the primary sialic acid moiety present in mammalian tissues, and sialic acid modification of human cell surfaces is used as a “self versus non-self” signal to the immune system, allowing for discrimination of cell types, among other functions of sialic acids [36]. In the eye, sialic acid is distributed in all structures, including cornea, sclera, AH, trabecular meshwork, lens, VH and retina, and its concentration seems to increase with aging [37]. SACOL0312 and SACOL0311 encode a putative sodium:solute symporter protein (NanT) and N-acetylneuraminate lyase (NanA), respectively. NanA converts N-acetylneuraminate to N-acetylmannosamine and pyruvate [38]. Recently, it was shown that *nanA* and *nanT* are co-transcribed in *S. aureus* strain AH1263 [30]. Further, *nanA* and *nanT* are required for *S. aureus* growth with sialic acid [30]. Our microarray results suggest that *S. aureus* SA564 transports and catabolizes sialic acid during growth in bovine AH.

Ascorbate (vitamin C) is abundant in the eye and present at much higher concentrations than in the plasma [39]. It is actively transported by the iris-ciliary body into the AH and serves as an antioxidant to protect the eye against light-induced free radical damage [40]. SACOL0400-SACOL0403 encode a putative ascorbate uptake transport system (*ulaABC*) and a predicted transcriptional antiterminator (Table 2 and Table S1). In *E. coli*, the PTS-like UlaABC system (alternatively named SgaTBA) transports ascorbate with concomitant phosphorylation, trapping ascorbate-6-phosphate in the cell [41]. *S. aureus* may transport ascorbate to prevent Fenton reaction damage occurring as a result of extracellular iron reduction by ascorbate under aerobic conditions [42].

Several virulence factors were specifically up-regulated during growth in AH, including an enterotoxin (*sec3*), the toxic shock syndrome toxin (*tst*), and a phenol soluble modulin (Table 2). The toxic shock syndrome toxin and the SEC enterotoxin are superantigens that are important in infections such as infective endocarditis and pneumonia [43]–[45]. Immunization against those exotoxins was found to protect against those serious illnesses [44], [46]. Phenol soluble modulins have also been found in animal models to have an essential role in bacteremia and skin infections [47]. The AH-specific up-regulation of these factors may facilitate translocation of *S. aureus* or *S. aureus*-produced factors into the posterior chamber and/or retinal damage during *S. aureus* endophthalmitis.

As for the analysis of the *codY* mutant, our microarray results demonstrate that *codY* deletion impacts expression of metabolic and virulence genes in *S. aureus* SA564. However, genes affected by *codY* were not consistent across the two pooled AH samples used here, suggesting that BCAA became depleted from one of the samples. These data indicate that, *in vivo* in the AC, where BCAA are present and replenished by AH turnover continuously, CodY repression may limit virulence of *S. aureus* during early stages of infection when cell densities are likely to be low. Consistent with this proposal, deletion of *codY* enhanced virulence of *S. aureus* in a murine AC infection model, as assessed by retinal function measurements, degree of inflammation in the eye, and histological assessments of ocular tissue damage. The microarray results suggest a role for enterotoxin (*sec3*), the toxic shock syndrome toxin (*tst*), and a phenol soluble modulin in endophthalmitis progression. Collectively, these data suggest that CodY repression of its target genes limits *S. aureus* disease in the murine AC infection model.

In conclusion, we used novel *in vitro* and *in vivo* infection models to characterize the behavior of *S. aureus* during endophthalmitis, one a nutritional model utilizing bovine ocular fluids as media for *S. aureus* growth *ex vivo*, and one an *in vivo* infection model evaluating endophthalmitis progression after *S. aureus* injection into the murine AC. We identified metabolic pathways that may be important for *S. aureus* endophthalmitis, specifically sialic acid, ascorbate, and pseudouridine metabolism. We are now directly assessing the roles of these pathways in the pathogenesis of *S. aureus* endophthalmitis. We additionally identified several virulence factors whose expression was activated by growth in ocular fluids, suggesting that transcriptional regulation of these genes is influenced by specific nutrients present in the eye. Our *in vivo* endophthalmitis model, a murine AC infection model, revealed a link between the BCAA-responsive transcriptional regulator CodY and experimental endophthalmitis progression. More specifically, relief of CodY repression of its target genes (by deletion of *codY*) enhanced *S. aureus* pathogenesis in the murine eye. Interestingly, this result suggests that it may be possible to use BCAA to mitigate *S. aureus* endophthalmitis progression by supplementing BCAA in eye drops postoperatively or by adding BCAA to the infusion solution that goes through the eye during intraocular surgery.

## Supporting Information

Table S1 **An expanded version of** **Table 2** **with probe set IDs, BLAST hit distribution among** ***S. aureus*** **COL, Mu50, N315, and NCTC 8325 genomes, and fold change data for every gene shown in** **Table 2** **, irrespective of meeting the fold change cut-off of 10.**
(PDF)

Table S2 **Expression data for all differentially expressed genes for the SA564 and SA564** ***codY*** **mutant during growth in CDM versus AH.**
(PDF)
