# Supplementary material for: In Vitro and In Vivo Models of Staphylococcus aureus Endophthalmitis Implicate Specific Nutrients in Ocular Infection
Source: PLoS One. 2014 Oct 23;9(10):e110872. doi: 10.1371/journal.pone.0110872 (PMC4207797; doi:10.1371/journal.pone.0110872)
Supplement: Table S1 — An expanded version of Table 2 with probe set IDs, BLAST hit distribution among S. aureus COL, Mu50, N315, and NCTC 8325 genomes, and fold change data for every gene shown in Table 2 , irrespective of meeting the fold change cut-off of 10. (PDF) [file pone.0110872.s001.pdf]

From Table 2 of manuscript:

Gene expression data not meeting > 10-fold cut-off is shown here in red.

NC = No change in > 1 of 4 comparisons

Presence/absence by BLAST analysis*:

*Best discontinuous megablast hits: for present genes (indicated by black square), probe sequence was present in the genome with at least 99% coverage and at least 96% sequence identity.

ORF	Gene	Description of gene or queried region	AH vs DM Fold regulation	VH vs DM Fold regulation	VH vs AH Fold regulation	DM1 vs DM2 Fold regulation	Probe ID	COL	Mu50	N315	NCTC 8325	discontinuous megaBLAST comments
SACOL0154	*aldA1*	Aldehyde dehydrogenase	13.2 (3.3)	5.0 (1.1)	NC		sa_c2918s2481_a_at	■	■	■	■	
SACOL0173	*ipdC*	Indole-3-pyruvate decarboxylase	12.1 (2.2)	12.1 (1.3)	1.0 (2.3)		sa_c3651s3133_a_at	■	■	■	■	
SACOL0176		Conserved hypothetical protein	42.2 (1.1)	3.9 (1.3)	-10.9 (1.3)		sa_c3778s3248_a_at	■	■	■	■	
SACOL0177		Glucokinase regulator-related protein	27.4 (1.2)	NC	-11.5 (1.2)		sa_c3814s3284_a_at	■	■	■	■	
SACOL0178*		PTS system, IIBC components (queries COL positions 205159-206204; overlaps probe set sa_c3850s3320_a_at)	21.1 (1.2)	2.3 (1.3)	-8.3 (1.3)		sa_i9845dr_x_at	■	■	■	■	
SACOL0178*		PTS system, IIBC components (queries COL positions 205325-206599; overlaps probe set sa_i9845dr_x_at)	17.8 (1.1)	2.1 (1.3)	-8.6 (1.4)		sa_c3850s3320_a_at	■	■	■	■	
SACOL0179		Phosphosugar-binding transcriptional regulator, RpiR family	12.1 (1.2)	NC	-6.2 (1.5)		sa_c3928s3395_a_at	■	■	■	■	
SACOL0192		Maltose ABC transporter, ATP-binding protein, putative	21.5 (1.5)	2.9 (1.1)	-6.5 (1.6)		sa_c5361s4637_a_at	■	■	■	■	
SACOL0193		Maltose ABC transporter, maltose-binding protein, putative	14.4 (1.3)	3.4 (1.2)	-4.2 (1.4)		sa_c4193s3545_a_at	■	■	■	■	
SACOL0194		Maltose ABC transporter permease protein	11.1 (1.5)	3.3 (1.4)	-3.7 (1.4)		sa_c4233s3585_a_at	■	■	■	■	
SACOL0195		Maltose ABC transporter permease protein	13.9 (1.5)	3.7 (1.4)	-3.4 (1.5)		sa_c10368s9029_a_at	■	■	■	■	
SACOL0196		Oxidoreductase, Gfo/Idh/MocA family	12.6 (1.5)	3.3 (1.3)	-3.9 (1.5)		sa_c4349s3699_a_at	■	■	■	■	
SACOL0197		Oxidoreductase, Gfo/Idh/MocA family	10.2 (1.4)	3.2 (1.1)	-3.2 (1.4)		sa_c4387s3735_a_at	■	■	■	■	
SACOL0198		Conserved hypothetical protein	10.7 (1.4)	3.1 (1.3)	-3.1 (1.2)		sa_c4410s3755_a_at	■	■	■	■	
SACOL0200*		Phosphoglycerate transporter family protein (queries 5' region of gene; COL positions 234237-234922)	44.5 (1.4)	NC	-27.9 (1.6)		sa_c10684s11134cv_s_at	■	■	■	■	
SACOL0200*		Phosphoglycerate transporter family protein (queries 3' region of gene; COL positions 234975-235574)	18.7 (1.8)	NC	-18.1 (1.8)		sa_c10685s11135_s_at	■	■	■	■	
SACOL0204	*pflB*	Formate acetyltransferase	16.0 (2.7)	NC	-4.4 (2.0)		sa_c9250s8097_a_at	■	■	■	■	
SACOL0205	*pflA*	Pyruvate formate-lyase-activating enzyme	11.5 (2.5)	4.3 (1.8)	-2.7 (2.0)		sa_c4619s3943_a_at	■	■	■	■	
SACOL0215		Propionate CoA-transferase, putative	NC (AH sample 1); 158-169X up-reg (AH sample 2)	10.7 (1.4)	-1.4 (17.4) (reflecting variable expression in 2 AH samples)		sa_c4917s4223_a_at	■	■	■	■	
SACOL0247	*lrgA*	Holin-like protein LrgA	7.0 (3.8)	NC	-14.4 (4.0)		sa_c6230s5410_at	■	■	■	■	
SACOL0248	*lrgB*	LrgB protein	6.3 (4.1)	NC	-11.1 (3.8)		sa_c6266s5446_a_at	■	■	■	■	
SACOL0308*	*[yeiC]*	Carbohydrate kinase, PfkB family (queries 5' region of gene; COL positions 342187-342850)	54.8 (1.5)	NC	-40.1 (1.8)		sa_c2469s9369_a_at	■	■	■	■	
SACOL0308*	*[yeiN]*	Carbohydrate kinase, PfkB family (queries 3' region of gene; COL positions 343019-343290)	34.3 (1.8)	NC	-36.1 (1.6)		sa_c10259s10671cv_s_at	■	■	■	■	
SACOL0309	*[yeiM]*	Conserved hypothetical protein	45.3 (1.6)	NC	-38.7 (1.7)		sa_c6947s6071_a_at	■	■	■	■	
SACOL0310		Nucleoside permease NupC, putative	28.3 (2.1)	NC	-32.0 (1.7)		sa_c6953s6075_a_at	■	■	■	■	
SACOL0311		Sodium:solute symporter family protein	38.1 (1.7)	NC	-13.2 (1.7)		sa_c6957s6079_a_at	■	■	■	■	
SACOL0312	*nanA*	N-acetylneuraminate lyase	54.8 (1.7)	3.5 (1.3)	-14.4 (1.7)		sa_c6959s6083_a_at	■	■	■	■	
SACOL0325		Prophage L54a, antirepressor, putative	-23.8 (2.2)	-9.9 (2.0)	NC		sa_c10438s10875_a_at	■	■		■	
SACOL0326		Hypothetical protein	-26.4 (2.4)	-15.7 (2.2)	NC		sa_c7158s10128_a_at	■	■	■	■	
SACOL0400	*[ulaA]*	Transport protein SgaT, putative	13.7 (1.4)	NC	-12.6 (1.4)		sa_c7008s6130_a_at	■	■	■	■	
SACOL0401	*[ulaB]*	Conserved hypothetical protein	20.7 (1.4)	NC	-16.0 (1.5)		sa_c10261s8939_a_at	■	■	■	■	
SACOL0402	*[ulaC]*	PTS system, IIA component	29.3 (1.4)	NC	-23.8 (1.4)		sa_c7012s6133_a_at	■	■	■	■	
SACOL0403		Transcriptional antiterminator, BglG family	28.3 (1.4)	NC	-28.3 (1.6)		sa_c7018s6139_a_at	■	■	■	■	
ig_SACOL0913		Intergenic region downstream of SACOL0913	-15.2 (1.9)	-6.3 (2.4)	NC		sa_i7487d_x_at	■	■	■	■	
SACOL0960	*rocD2*	Ornithine aminotransferase	10.0 (2.0)	3.5 (1.2)	-2.9 (2.1)		sa_c8673s7622_a_at	■	■	■	■	
SACOL1187		Antibacterial protein (phenol soluble modulin)	10.4 (2.5)	NC	-6.5 (1.8)		sa_c1063s849_at	■	■	■	■	
SACOL1360		Aspartate kinase	10.6 (5.9)	6.0 (1.3)	-1.6 (6.1)		sa_c9581s8342_a_at	■	■	■	■	
SACOL1734	*gapA2*	Glyceraldehyde-3-phosphate dehydrogenase	10.4 (4.9)	NC	-6.8 (4.9)		sa_c3059s2614_a_at	■	■	■	■	
SACOL1784	*acuA*	Acetoin utilization protein AcuA	13.7 (3.6)	NC	NC		sa_c3244s2793_a_at	■	■	■	■	
SACOL1785	*acuC*	Acetoin utilization protein AcuC	10.6 (3.2)	NC	NC		sa_c3250s2795_a_at	■	■	■	■	
SACOL1816	*putA*	Proline dehydrogenase	21.1 (1.7)	3.7 (1.4)	-5.4 (1.7)		sa_c3376s2908_a_at	■	■	■	■	
SACOL2163		Conserved hypothetical protein	11.7 (1.4)	7.2 (1.5)	NC		sa_c4631s9238_a_at	■	■	■	■	
SACOL2247		Hypothetical protein	-19.7 (7)	NC	NC		sa_i10628d_x_at	■	■	■	■	
SACOL2356		ABC transporter, ATP-binding protein	2.8 (3.8)	NC	-10.0 (2.7)		sa_c5303s4583_a_at	■	■	■	■	
SACOL2357		ABC transporter, permease protein	2.3 (3.9)	NC	-10.6 (2.9)		sa_c5307s4587_at	■	■	■	■	
SACOL2403		ABC transporter, substrate binding protein	-6.7 (1.5)	-15.7 (1.5)	-2.1 (1.3)		sa_c5608s4864_a_at	■	■	■	■	
SACOL2415	*gpm*	Phosphoglycerate mutase	10.9 (1.7)	12.1 (1.6)	NC		sa_c5644s4894_a_at	■	■	■	■	
SACOL2427	*bioA*	Adenosylmethionine-8-amino-7-oxononanoate aminotransferase	8.3 (3.5)	13.9 (1.5)	1.6 (3.5)		sa_c5690s4940_a_at	■	■	■	■	
SACOL2428	*bioD*	Dethiobiotin synthase	16.0 (3.9)	24.3 (1.7)	NC		sa_c5696s9156_a_at	■	■	■	■	
SACOL2441		Amino acid permease	10.6 (4.4)	3.9 (1.7)	NC		sa_c9348s9368_a_at	■	■	■	■	
SACOL2509	*fnbB*	Fibronectin binding protein B	-4.4 (1.4)	-13.0 (1.3)	-3.0 (1.5)		sa_c9369s8195_a_at	■			■	Hits to Mu50 and N315: 100% sequence coverage, 83% nucleotide iden
SACOL2527		Fructose-1,6-bisphosphatase, putative	11.3 (2)	6.7 (1.2)	NC		sa_c6036s5235_a_at	■	■	■	■	
SACOL2554_1	*cidA*	LrgA family protein	-2.7 (1.3)	12.8 (1.4)	34.9 (1.4)		sa_c6135s5319_a_at	■	■	■	■	
SACOL2652**	*clfB* rev comp	Queries 97 nt region complementary to 5' end of *clfB* (clumping factor B); COL positions 2713662-2713758)	-30.4 (1.2)	-6.4 (2.0)	NC		sa_c9373s10359_a_at	■	■	■	■	
SACOL2694	*geh*	Lipase	19.4 (1.4)	3.7 (1.3)	-4.4 (1.5)		sa_c6688s5833_a_at	■	■	■	■	
SAOUHSC_02028 *		phiETA ORF57-like protein (queries NCTC 8325 positions 1932884-1931778; overlaps probe set sa_c10392s10798_at)	-22.6 (1.9)	-25.5 (1.4)	NC		sa_c10392s10798_s_at				■	Hit to Mu50: 100% sequence coverage, 80% nucleotide identity
SAOUHSC_02028 *		phiETA ORF57-like protein (queries NCTC 8325 positions 1932687-1931115; overlaps probe set sa_c10392s10798_s_at)	-9.5 (1.2)	-16.9 (1.8)	NC		sa_c10392s10798_at				■	Hit to Mu50: 99% sequence coverage, 80% nucleotide identity
SAOUHSC_02078		phi PV83 orf 10-like protein	-20.0 (2.0)	-10.4 (1.8)	NC		sa_c10437s10872_a_at				■	
SAOUHSC_02206		Hypothetical phage protein	-10.4 (1.6)	-9.5 (1.6)	NC		sa_c10456s10893_a_at				■	
SAV0859		Hypothetical protein	-17.1 (1.7)	-8.0 (2.3)	NC		sa_c10436s10870_a_at		■		■	
ig_SAV0860		Intergenic region downstream of SAV0860	-14.2 (1.8)	-10.7 (1.9)	NC		sa_c10023s10486_a_at		■			
SAV0905		Similar to phiETA ORF57-like protein	-22.6 (1.9)	-25.5 (1.4)	NC		sa_c10393s10799_s_at		■			Hit to NCTC 8325: 100% sequence coverage, 82% nucleotide identity
SAV1985		Hypothetical protein	-17.4 (1.9)	-11.5 (2.0)	NC		sa_c4057s9934_a_at		■			Hit to N315: 79% sequence coverage, 70% nucleotide identity
SA1817	*sec3*	Enterotoxin type C3	10.9 (3.0)	NC	-6.9 (2.4)		sa_c3559s9825_a_at		■	■		Hit to COL: 99% sequence coverage, 73% nucleotide identity
SA1819	*tst*	Toxic shock syndrome toxin-1	15.2 (5.5)	NC	-8.3 (5.7)		sa_c10149s10564_at		■	■		

ORF	Gene	PsortB final prediction	PsortB internal helices detected	PsortB signal peptide detected	Selected BLASTP hit (% identity, % similarity, e-value; % query sequence aligned)	Pfam hit (e-value)	Pfam hit description (from www.pfam.org)
SACOL0154	*aldA1*	Cytoplasmic	0	no		Aldedh (2e-172)	Family of dehydrogenases that act on aldehyde substrates; use NADP as cofactor
SACOL0173	*ipdC*	Cytoplasmic Membrane	0	no		TPP_enzyme_N (4.2e-36); TPP_enzyme_M (3.4e-36); TPP_enzyme_C (8.5e-16)	Thiamine pyrophosphate enzyme
SACOL0176		Cytoplasmic	0	no		DUF871 (4.3e-107)	Function unknown
SACOL0177		Cytoplasmic	0	no		SIS_2 (1.4e-07)	Sugar isomerase domain
SACOL0178*		Cytoplasmic Membrane	10	no		PTS_EIIB (1.2e-14); PTS_EIIC (9.8e-31)	Phosphotransferase system
SACOL0178*		Cytoplasmic Membrane	10	no		PTS_EIIB (1.2e-14); PTS_EIIC (9.8e-31)	Phosphotransferase system
SACOL0179		Cytoplasmic	0	no		HTH_6 (1.8e-22); SIS (4e-15)	Helix-turn-helix DNA binding domain, RpiR family; Sugar isomerase domain
SACOL0192		Cytoplasmic Membrane	0	no		ABC_tran (2.6e-22); TOBE (1.4e-08)	ABC transporter; Transport-associated oligonucleotide/oligosaccharide binding
SACOL0193		Unknown	0	yes		SBP_bac_8 (2e-39)	Bacterial extracellular solute-binding protein
SACOL0194		Cytoplasmic Membrane	8	no		TM2 (8.6e-08); BPD_transp_1 (6e-26)	Family composed of a pair of transmembrane alpha helices connected by a short linker; binding-protein-dependent transport system inner membrane component
SACOL0195		Cytoplasmic Membrane	6	no		BDP_transp_1 (2.1e-18)	Binding-protein-dependent transport system inner membrane component
SACOL0196		Cytoplasmic	0	no		GFO_IDH_MocA (4.1e-31); GFO_IDH_MocA_C (1.5e-04)	Oxidoreductase family, NAD-binding Rossmann fold and C-terminal alpha/beta domain
SACOL0197		Cytoplasmic	0	no		GFO_IDH_MocA (2.1e-30); GFO_IDH_MocA_C (2.5e-08)	Oxidoreductase family, NAD-binding Rossmann fold and C-terminal alpha/beta domain
SACOL0198		Cytoplasmic	0	no		AP_endonuc_2 (4.2e-33); AP_endonuc_2_N (1.4e-20)	Xylose isomerase-like TIM barrel (present in endonuclease IV); AP endonuclease family 2 C terminus, highly-conserved sequence found at the C terminus of several apurinic/apyrimidinic (AP) endonucleases, in a range of Gram-positive and Gram-negative bacteria
SACOL0200*		Cytoplasmic Membrane	12	no		MFS_1 (1e-39)	Major facilitator superfamily transporter
SACOL0200*		Cytoplasmic Membrane	12	no		MFS_1 (1e-39)	Major facilitator superfamily transporter
SACOL0204	*pflB*	Cytoplasmic	0	no		PFL (4.9e-209); Gly_radical (4.3e-43)	Pyruvate formate lyase; glycine radical
SACOL0205	*pflA*	Cytoplasmic	0	no		Fer4_12 (3.1e-19); Radical_SAM (3.9e-27)	4Fe-4S single cluster domain; radical SAM superfamily
SACOL0215		Unknown	0	no		CoA_trans (1.4e-19)	Coenzyme A transferase
SACOL0247	*lrgA*	Cytoplasmic Membrane	4	no		LrgA (2.1e-25)	Uncharacterized family containing LrgA, thought to export murein hydrolases
SACOL0248	*lrgB*	Cytoplasmic Membrane	7	no		LrgB (6.1e-76)	LrgB-like family
SACOL0308*	*[yeiC]*	Cytoplasmic	0	no	YeiC, Escherichia coli UTI89 (29%, 48%, 3e-31; 78	HTH_24 (8.3e-10); PfkB (1.7e-40)	Winged helix-turn-helix DNA-binding; family includes a variety of carbohydrate and pyrimidine kinases
SACOL0308*	*[yeiN]*	Cytoplasmic	0	no	YeiN, Escherichia coli UTI89 (57%, 73%, 2e-115; 9	HTH_24 (8.3e-10); PfkB (1.7e-40)	Winged helix-turn-helix DNA-binding; family includes a variety of carbohydrate and pyrimidine kinases
SACOL0309	*[yeiM]*	Cytoplasmic	1	no	YeiM, Escherichia coli UTI89 (31%, 54%, 9e-57; 99	Indigoidine_A (2.9e-135)	pseudouridine-5'-phosphate glycosidase
SACOL0310		Cytoplasmic Membrane	9	no		Nucleos_tra2_N (1.9e-18); Nucleos_tra2_C (1.8e-64)	Na+ dependent nucleoside transporter
SACOL0311		Cytoplasmic Membrane	12	no		SSF (8e-41)	Sodium-solute symporter
SACOL0312	*nanA*	Cytoplasmic	0	no		DHDPS (5.6e-79)	Dihydrodipicolinate synthase (N-acetylneuraminate lyase included in this family)
SACOL0325		Cytoplasmic	0	no		Bro-N (1.1e-27); ANT (1.6e-31)	: Phage antirepressor protein KilAC domain
SACOL0326		Unknown	0	no		no hits	--
SACOL0400	*[ulaA]*	Cytoplasmic Membrane	12	no	UlaA, Escherichia coli MG1655 (28%, 52%, 6e-41;	EIIC-GAT (7.8e-137)	PTS system sugar-specific permease component
SACOL0401	*[ulaB]*	Cytoplasmic	0	no	UlaB, Escherichia coli MG1655 (30%, 49%, 1e-04,	PTS_IIB (1.4e-13)	Phosphotransferase system
SACOL0402	*[ulaC]*	Cytoplasmic	0	no	UlaC, Escherichia coli MG1655 (27%, 53%, 1e-18,	PTS_EIIA_2 (6.8e-31)	Phosphotransferase system
SACOL0403		Cytoplasmic	0	no		HTH_11 (2.2e-07); HTH_11 (1.9e-08); PRD (9.8e-16); PRD (4.8e-22); PTS_EIIA_2 (6.4e-18)	
ig_SACOL0913		--	--	--		--	--
SACOL0960	*rocD2*	Cytoplasmic	0	no		Aminotran_3 (1e-114)	Aminotransferase class-III
SACOL1187		Unknown	0	no		Staph_haemo (2.6e-24)	Staphylococcus haemolytic protein
SACOL1360		Cytoplasmic	0	no		AA_kinase (5.8e-42); ACT_7 (1.2e-05)	
SACOL1734	*gapA2*	Cytoplasmic	0	no		Gp_dh_N (6e-51); Gp_dh_C (2.7e-65)	
SACOL1784	*acuA*	Cytoplasmic	0	no		--	--
SACOL1785	*acuC*	Cytoplasmic	0	no		Hist_deacetyl (1.2e-72)	Histone deacetylase domain; includes acetoin utilization proteins
SACOL1816	*putA*	Cytoplasmic	0	no		Pro_dh (6.7e-56)	
SACOL2163		Unknown	0	no		--	--
SACOL2247		Unknown	0	no		--	--
SACOL2356		Cytoplasmic Membrane	0	no		ABC_tran (1.2e-25)	ABC transporter
SACOL2357		Cytoplasmic Membrane	4	no		MacB_PCD (7.8e-16); FtsX (1.8e-10)	
SACOL2403		Cytoplasmic Membrane	0	yes		SBP_bac_9 (1.2e-63); YodA (2.2e-84)	
SACOL2415	*gpm*	Unknown	0	no		His_Phos_1 (2.1e-41)	
SACOL2427	*bioA*	Cytoplasmic	0	no		Aminotran_3 (3.2e-107)	Aminotransferase class-III
SACOL2428	*bioD*	Cytoplasmic	0	no		AAA_26 (7e-25)	ATPases Associated with diverse cellular Activities, found in cofactor biosynthesis proteins such as dethiobiotin synthase and cobyric acid synthase
SACOL2441		Cytoplasmic Membrane	12	no		AA_permease (1e-96)	
SACOL2509	*fnbB*	Cell wall	0	yes		YSIRK_signal (1.4e-07); SdrG_C_C (7.3e-42); Fn_bind (4e-10); Fn_bind (4.9e-11); Fn_bind (5.1e-11); Gram_pos_anchor (1.8e-07)	
SACOL2527		Cytoplasmic	0	no		FBPase_2 (1.7e-310)	
SACOL2554_1	*(cidA)*	Cytoplasmic Membrane	4	no		LrgA (1.9e-25)	Uncharacterized family containing LrgA, thought to export murein hydrolases
SACOL2652**	*clfB* rev comp	--	--	--		--	--
SACOL2694	*geh*	Extracellular	1	yes		YSIRK_signal (4.4e-12); Abhydrolase_5 (1e-03)	
SAOUHSC_02028*		Unknown	0	no		DUF2479 (2.6e-56)	Found in phage from a number of bacteria
SAOUHSC_02028*		Unknown	0	no		DUF2479 (2.6e-56)	Found in phage from a number of bacteria
SAOUHSC_02078		Unknown	0	no		none	--
SAOUHSC_02206		Cytoplasmic	0	no		none	--
SAV0859		Cytoplasmic	0	no		none	--
ig_SAV0860		--	--	--		--	--
SAV0905		Unknown	0	no		DUF2479 (2.5e-57)	Found in phage from a number of bacteria
SAV1985		Unknown	0	no		RecT (9.7e-52)	Includes proteins related to RecT, a DNA single-strand annealing protein
SA1817	*sec3*	Extracellular	1	yes		Stap_Strp_toxin (1.9e-28); Stap_Strp_tox_C (9.4e-34)	Staphylococcal/Streptococcal toxin, OB-fold domain; Staphylococcal/Streptococcal toxin, beta-grasp domain
SA1819	*tst*	Extracellular	0	yes		Stap_Strp_toxin (2.7e-13); Stap_Strp_tox_C (1e-09)	Staphylococcal/Streptococcal toxin, OB-fold domain; Staphylococcal/Streptococcal toxin, beta-grasp domain

Probe sets not shown in manuscript:

Presence/absence by BLAST analysis:

ORF	Gene	Description of gene or queried region	AH vs DM Fold regulation**	VH vs DM Fold regulation	VH vs AH Fold regulation		Probe ID	COL	Mu50	N315	NCTC 8325
			8.0 (2.2)	11.1 (2.4)	NC		sa_i7808d_x_at	■	■	■	■
			8.9 (2.5)	10.4 (2.5)	NC		sa_i9119u10r_x_at	■	■	■	■
SACOL0100		Cysteine synthase/cystathionine β-synthase family protein		12.3 (7)		-30	sa_c9525s8305_a_at	■	■	■	■
SACOL0101		Ornithine cyclodeaminase, putative		11.9 (6.4)		-27.9	sa_c1308s1080_a_at	■	■	■	■
SACOL0102	*lucC*	Siderophore biosynthesis protein, IucC family		13.2 (6.7)		-30	sa_c1340s1111_a_at	■	■	■	■
SACOL0103		Drug transporter, putative		15.7 (5.9)		-22.6	sa_c1358s1133_a_at	■	■	■	■
SACOL0104		Siderophore biosynthesis protein, IucA family		11.3 (5.4)		-19.7	sa_c1388s1161_a_at	■	■	■	■
SACOL0108		Hypothetical protein		11.3 (3.9)		-11.3	sa_c1546s1320_a_at	■	■	■	■
SACOL0688		ABC transporter, substrate-binding protein		14.9 (3.1)		-7	sa_c8892s7808_a_at	■	■	■	■
SACOL0689		ABC transporter, permease protein		13.9 (4.4)		-13	sa_c7949s6934_a_at	■	■	■	■
SACOL0690		ABC transporter, ATP-binding protein		14.9 (4.5)		-13	sa_c5408s4676_a_at	■	■	■	■
SACOL1138	*sasJ /isdB*	LPXTG cell wall surface anchor protein		16.3 (5.5)		-18.4	sa_c896s691_a_at	■	■	■	■
SACOL2021-2	RNAIII (partial)	3' 345 nt of RNAIII; probes region downstream of δ-hemolysin gene	10.4 (3.5)	12.3 (3.7)		-7.5	sa_i8854u_x_at	■	■	■	■
