# Supplementary material for: In Vitro and In Vivo Models of Staphylococcus aureus Endophthalmitis Implicate Specific Nutrients in Ocular Infection
Source: PLoS One. 2014 Oct 23;9(10):e110872. doi: 10.1371/journal.pone.0110872 (PMC4207797; doi:10.1371/journal.pone.0110872)
Supplement: Table S2 — Expression data for all differentially expressed genes for the SA564 and SA564 codY mutant during growth in CDM versus AH. (PDF) [file pone.0110872.s002.pdf]

From Table 3 of manuscript:

ORF	Gene	Description of gene or queried region	CDM7 (*codY* mutant) versus SA564: DM Fold regulation	AH Fold regulation	VH Fold regulation	Average fold regulation, AH vs CDM	Fold change - AH1 versus CDM1	Fold change - AH1 versus CDM2	Fold change - AH2 versus CDM1	Fold change - AH2 versus CDM2	Probe ID	Presence/absence by BLAST analysis*: COL	Mu50	N315	NCTC 8325	BLAST comments
SA1817	*sec3*	enterotoxin type C3	6.7 (1.2)			15.2 (5.5)	26.0	29.9	4.0	4.6	sa c3559s9825 a at		■	■		Hit to COL: 99% sequence coverage, 73% nucleotide identity
SACOL0136	*cap5A*	capsular polysaccharide biosynthesis protein Cap5A	8.0 (1.6)			5.8 (1.6)	9.2	7.5	4.6	3.5	sa c2346s1974 a at	■	■	■	■	
SACOL0138	*cap5C*	capsular polysaccharide biosynthesis protein Cap5C	6.2 (1.9)			NC	9.2	5.3	3.2	NC	sa c2399s1991 a at	■	■	■	■	
SACOL0185		peptide ABC transporter permease	53.8 (1.3)	6.5 (1.9)	11.5 (1.2)	4.8 (1.7)	2.6	3.7	6.5	8.0	sa c4016s3424 a at	■	■	■	■	
SACOL0186		peptide ABC transporter permease	31.5 (1.2)	5.9 (1.9)	10.9 (1.1)	NC	NC	NC	4.6	4.0	sa c4034s3428 a at	■	■	■	■	
SACOL0187		RGD-containing lipoprotein	29.3 (1.3)		11.9 (1.1)	2.8 (1.9)	1.4	2.0	3.7	5.7	sa c4055s3432 a at	■	■	■	■	
SACOL0188	*gat*	gamma-glutamyltranspeptidase	13.0 (1.2)		7.1 (1.1)	NC	NC	NC	2.1	2.1	sa c4081s3441 a at	■	■	■	■	
SACOL0214		long-chain-fatty-acid--CoA ligase			6.3 (2.4)	NC	NC	NC	111.4	119.4	sa c4884s4191 a at	■	■	■	■	
SACOL0215		propionate CoA-transferase			8.4 (1.5)	NC	NC	NC	157.6	168.9	sa c4917s4223 a at	■	■	■	■	
SACOL0222	*ldh1*	L-lactate dehydrogenase	7.9 (2.2)			-1.0 (9.8)	12.1	3.0	-3.0	-13.9	sa c5235s4535 a at	■	■	■	■	
SACOL0267		hypothetical protein	7.2 (1.0)			3.0 (2.2)	1.5	1.5	6.1	6.1	sa c6853s5989 a at	■	■	■	■	
SACOL0270		staphyloxanthin biosynthesis protein	6.5 (1.4)			2.2 (1.5)	1.3	2.8	1.9	3.5	sa c6859s5993 a at	■	■	■	■	
SACOL0271		hypothetical protein	5.3 (1.1)			2.1 (1.3)	1.6	1.7	2.5	2.8	sa c9261s8106 at	■	■	■	■	
SACOL0274		Hypothetical protein	5.0 (3.2)			NC	NC	NC	1.5	14.9	sa c10505s9038 a at	■	■	■	■	
SACOL0308***		Carbohydrate kinase (3' region)		-7.2 (1.7)		34.3 (1.8)	19.7	22.6	55.7	55.7	sa c10259s10671cv s at	■	■	■	■	
SACOL0308***		carbohydrate kinase (5' region)		-6.0 (1.6)		54.8 (1.5)	34.3	45.3	78.8	73.5	sa c2469s9369 a at	■	■	■	■	
SACOL0309		hypothetical protein		-6.8 (1.7)		45.3 (1.6)	27.9	32.0	68.6	68.6	sa c6947s6071 a at	■	■	■	■	
SACOL0310		nucleoside permease NupC		-7.2 (2.0)		28.3 (2.1)	29.9	10.6	64.0	32.0	sa c6953s6075 a at	■	■	■	■	
SACOL0427		hypothetical protein	7.5 (1.1)			1.6 (1.1)	1.5	1.4	1.7	1.6	sa c7094s6206 a at	■	■	■	■	
SACOL0428	*metE*	5-methyltetrahydropteroyltriglutamate/homocysteine S-methyltransferase	19.7 (1.2)		9.2 (1.6)	4.7 (1.4)	4.0	3.2	6.5	5.7	sa c7100s6210 a at	■	■	■	■	
SACOL0429		bifunctional homocysteine S-methyltransferase/5,10-methylenetetrahydrofolate reductase	28.3 (1.2)		11.5 (1.4)	4.4 (2.2)	2.5	2.1	9.2	8.0	sa c7102s6213 a at	■	■	■	■	
SACOL0430		trans-sulfuration enzyme family protein	36.1 (1.1)		12.8 (1.1)	4.5 (2.9)	1.7	1.9	10.6	12.1	sa c7106s6219 a at	■	■	■	■	
SACOL0431		trans-sulfuration enzyme family protein	30.9 (1.1)		10.4 (1.1)	3.3 (3.0)*	1.3	NC	9.2	7.5	sa c7112s6224 a at	■	■	■	■	
SACOL0431 rev comp		Reverse complement of interior 105 nt region of SACOL0431	11.1 (1.4)			NC	NC	NC	NC	NC	sa c7110s10118 at	■	■	■	■	
ig SACOL0431-2		121 bp region upstream of SACOL0431	12.3 (1.7)			NC	NC	NC	3.5	1.9	sa i6224u x at	■	■	■	■	
SACOL0480		hypothetical protein	9.7 (1.2)			3.6 (1.2)	4.6	4.0	3.2	2.8	sa c7313s9398 a at	■	■	■	■	
SACOL0502		cysteine synthase/cystathionine beta-synthase	7.6 (2.0)			NC	NC	3.5	-1.7	2.0	sa c7368s9209 a at	■	■	■	■	
SACOL0503***	*metB*	Cystathionine gamma-synthase	7.9 (2.1)			NC	NC	3.5	-2.5	1.4	sa c10721s11169cv s at	■	■	■	■	100% coverage, 100% nucleotide identity in Mu50 and N315. 100% coverage and 89% identity for COL and NCTC 8325. BLASTX hits are 100% identical for all four strains. So nucleotide sequence divergence in COL and NCTC 8325.
SACOL0503***	*metB*	Cystathionine gamma-synthase	7.1 (2.0)			NC	NC	3.0	-2.3	NC	sa c7372s10188cs s at	■	■	■	■	
SACOL0504		ABC transporter ATP-binding protein	30.4 (2.1)			NC	NC	2.6	4.0	13.0	sa c5418s4689 a at	■	■	■	■	
SACOL0505		ABC transporter permease	29.3 (2.4)			NC	NC	2.6	3.2	14.9	sa c7374s6406 a at	■	■	■	■	
SACOL0506		ABC transporter substrate-binding protein	18.4 (1.5)			NC	NC	1.6	2.6	4.9	sa c7378s6412 a at	■	■	■	■	
SACOL0514	*altB*	glutamate synthase	23.4 (1.3)		12.1 (1.5)	NC	NC	-1.9	NC	NC	sa c7410s6434 a at	■	■	■	■	
SACOL0515	*altD*	glutamate synthase, small subunit	15.7 (1.3)		9.8 (1.2)	NC	NC	-1.5	NC	NC	sa c7412s6438 a at	■	■	■	■	
ig_SACOL0701-2 rev comp	overlaps RsaD sRNA	reverse complement of intergenic region between SACOL0701-2; 106 5' nt overlaps RsaD sRNA from Geissmann, et al. 2009 Nucleic Acids Res	20.4 (2.1)	5.4 (1.4)	9.2 (1.4)	NC	NC	NC	3.7	NC	sa i6966ur x at	■	■	■	■	
SACOL0796		iron compound ABC transporter permease	-5.5 (2.3)			-1.2 (2.2)	-2.1	1.7	-2.6	1.4	sa c8276s7256 a at	■	■	■	■	
SACOL0797		iron compound ABC transporter permease	-5.8 (2.0)			NC	-2.0	1.6	-3.7	NC	sa c8283s7260 a at	■	■	■	■	
SACOL0798		iron compound ABC transporter ATP-binding protein	-6.0 (2.3)			NC	-2.0	1.9	-2.8	NC	sa c5353s4626 a at	■	■	■	■	
SACOL0815		ribosomal subunit interface protein	5.5 (1.7)			3.3 (1.7)	5.7	2.5	4.6	1.9	sa c8966s7880 a at	■	■	■	■	
SACOL0860	*nuc*	thermonuclease precursor	9.2 (1.2)		5.3 (1.3)	4.2 (1.5)	5.7	6.1	3.2	2.8	sa c8453s7413 a at	■	■	■	■	
SACOL0991	*oppB*	Oligopeptide ABC transport permease	10.7 (1.3)			NC	1.6	NC	16.0	13.9	sa c10571s9056 a at	■	■	■	■	
SACOL0992	*oppC*	oligopeptide ABC transporter permease	11.5 (1.4)			5.7 (4.1)	1.5	1.9	18.4	19.7	sa c324s166 a at	■	■	■	■	
SACOL0993	*oppD*	oligopeptide ABC transporter ATP-binding protein	8.3 (1.3)			3.8 (3.3)	1.2	1.5	9.2	12.1	sa c328s170 a at	■	■	■	■	
SACOL0994	*oppF*	oligopeptide ABC transporter ATP-binding protein	7.9 (1.2)			3.9 (3.0)	1.4	1.6	9.8	10.6	sa c332s172 a at	■	■	■	■	
SACOL0995		oligopeptide ABC transporter oligopeptide-binding protein	8.3 (1.2)			4.6 (2.7)	1.7	2.1	10.6	11.3	sa c350s191 a at	■	■	■	■	
SACOL1018		sodium:alanine symporter family protein	21.9 (1.2)	9.9 (3.4)	15.7 (1.7)	1.6 (3.3)	-1.4	-2.0	5.3	3.7	sa c434s266 a at	■	■	■	■	
ig SACOL1018-9		intergenic region between SACOL1018 and 1019		12.3 (1.7)	21.9 (3.7)	NC	NC	NC	NC	NC	sa i269u x at	■	■	■	■	
SACOL1019		hypothetical protein	6.1 (1.1)			NC	NC	NC	2.1	2.0	sa c439s269 a at	■	■	■	■	
SACOL1033		hypothetical protein	8.9 (1.5)			3.0 (1.6)	3.0	1.7	5.7	2.8	sa c485s314 at	■	■	■	■	
SACOL1038		Membrane protein	9.5 (1.2)	6.3 (2.1)	12.3 (1.3)	NC	NC	NC	4.9	6.5	sa c503s329 a at	■	■	■	■	
SACOL1039		hypothetical protein	7.0 (1.2)	5.6 (1.6)	13.0 (1.5)	NC	NC	NC	3.2	3.0	sa c507s335 a at	■	■	■	■	
SACOL1040		ABC transporter ATP-binding protein	9.4 (1.5)	7.6 (2.0)	8.4 (1.6)	NC	NC	NC	5.3	2.5	sa c515s343 a at	■	■	■	■	
SACOL1186		Anti protein (phenol soluble modulin)	6.8 (2.6)			7.5 (2.7)	3.7	19.7	2.8	14.9	sa c1059s845 at	■	■	■	■	Present but not annotated in Mu50 and N315
SACOL1187		Antibacterial protein (phenol soluble modulin)	6.8 (2.4)			10.4 (2.5)	5.7	27.9	4.0	18.4	sa c1063s849 at	■	■	■	■	Present but not annotated in Mu50 and N315
SACOL1272***	*codY*	transcriptional repressor CodY	-789.6 (1.1)	-652.6 (1.4)	-197.4 (1.5)	NC	NC	NC	NC	NC	sa c9313s8155 a at	■	■	■	■	
SACOL1272***	*codY*	transcriptional repressor CodY (overlaps region queried by c9313s8155 a at)	-260.5 (2.3)	-309.8 (1.3)	-207.9 (1.5)	NC	1.5	1.6	NC	NC	sa i11098dr x at	■	■	■	■	
SACOL1272 rev comp***		Reverse complement of codY (corresponds to 3' region of codY)	-40.1 (1.8)			-3.0 (3.6)	1.4	-1.7	-4.9	-13.9	sa c10645s11098 a at	■	■	■	■	
SACOL1272 rev comp***		Reverse complement of codY (corresponds to 5' region of codY)	-98.7 (2.0)	-56.7 (2.6)		NC	1.3	NC	-3.7	-8.6	sa i11098d x at	■	■	■	■	
SACOL1360		Aspartate kinase	30.4 (1.3)			10.6 (5.9)	2.8	1.9	55.7	42.2	sa c9581s8342 a at	■	■	■	■	
SACOL1362	*hom*	Homoserine dehydrogenase	16.3 (1.3)			8.0 (2.3)	3.7	4.0	16.0	17.1	sa c1659s1395 a at	■	■	■	■	
SACOL1363	*thrC*	Threonine synthase	17.5 (1.4)			9.0 (2.4)	4.0	4.6	18.4	19.7	sa c1665s1401 a at	■	■	■	■	
SACOL1364	*thrB*	Homoserine kinase	14.9 (1.3)			7.5 (2.3)	3.5	3.7	14.9	16.0	sa c1669s1406 a at	■	■	■	■	
SACOL1368	*katA*	catalase	5.3 (1.7)			3.5 (1.9)	4.0	1.6	8.0	3.0	sa c1679s1417 a at	■	■	■	■	
SACOL1403	*trpE*	Anthranilate synthase component I	6.0 (1.4)			NC	NC	NC	1.5	1.5	sa c1818s1543 a at	■	■	■	■	
SACOL1404	*trpG*	Anthranilate synthase component II	6.4 (1.5)			NC	-2.6	-2.0	NC	NC	sa c1820s1547 a at	■	■	■	■	
SACOL1405	*trpD*	Anthranilate phosphoribosyltransferase	8.6 (1.6)		6.5 (1.1)	NC	NC	NC	2.1	2.0	sa c1828s1551 a at	■	■	■	■	
SACOL1406	*trpC*	Indole-3-glycerol-phosphage synthase	13.7 (1.5)		7.7 (1.2)	NC	NC	NC	4.9	5.3	sa c1832s1558 a at	■	■	■	■	
SACOL1407	*trpF*	N-(5'-phosphoribosyl)anthranilate isomerase	21.9 (1.5)		10.0 (1.3)	NC	NC	NC	6.1	5.3	sa c1836s1562 at	■	■	■	■	
SACOL1408	*trpB*	tryptophan synthase subunit beta	18.4 (1.7)		6.7 (1.2)	NC	NC	NC	2.8	4.9	sa c1840s1566 a at	■	■	■	■	
SACOL1409	*trpA*	tryptophan synthase subunit alpha	6.1 (1.4)			NC	NC	NC	1.7	1.5	sa c1844s1570 a at	■	■	■	■	
SACOL1428	*lysC*	Aspartate kinase	10.9 (1.7)			NC	1.9	NC	13.9	6.1	sa c1912s1635 a at	■	■	■	■	
SACOL1429	*asd*	aspartate semialdehyde dehydrogenase	14.2 (1.4)			NC	2.1	NC	13.0	7.5	sa c1918s1640 a at	■	■	■	■	
SACOL1430	*dapA*	dihydrodipicolinate synthase	13.5 (1.2)			4.2 (2.6)	2.0	1.7	9.8	9.2	sa c1922s1644 a at	■	■	■	■	
SACOL1431	*dapB*	dihydrodipicolinate reductase	14.4 (1.2)			5.0 (2.4)	2.6	2.1	12.1	9.2	sa c1924s1648 a at	■	■	■	■	
SACOL1432	*dapD*	2,3,4,5-tetrahydropyridine-2,6-dicarboxylate N-succinyltransferase	13.0 (1.1)			4.8 (1.8)	3.0	2.6	8.6	7.5	sa c1928s1652 a at	■	■	■	■	
ig SACOL1432-3		intergenic region between dapD and SACOL1433	26.4 (1.8)			NC	NC	4.6	21.1	14.9	sa i1655u x at	■	■	■	■	
SACOL1433		M20/M25/M40 family peptidase	9.2 (1.3)			NC	1.9	NC	7.5	5.3	sa c1934s1655 a at	■	■	■	■	
SACOL1434		Alanine racemase	9.2 (1.2)			3.7 (1.9)	2.3	2.0	7.0	5.7	sa c1936s1659 a at	■	■	■	■	
SACOL1449	*sucA*	2-oxoglutarate dehydrogenase E1 component	5.2 (1.1)			9.7 (1.5)	7.0	6.5	14.9	13.0	sa c1994s9145 a at	■	■	■	■	
SACOL1772		Class V aminotransferase	24.3 (1.2)	5.4 (2.9)	13.7 (1.2)	NC	NC	NC	6.5	6.1	sa c3202s2750 a at	■	■	■	■	
SACOL1773	*serA*	D-3-phosphoglycerate dehydrogenase	21.5 (1.3)		11.9 (1.2)	NC	1.7	NC	8.0	4.9	sa c3204s2753 a at	■	■	■	■	
SACOL2003	*hlb*	Queries 170 nt (positions 19-188) in 5' region of hlb (phospholipase C)	5.6 (1.3)			NC	21.1	26.0	NC	NC	sa c3951s9849 a at	■	■	■	■	
SACOL2021-2	RNAIII (partial)	3' 345 nt of RNAIII; probes region downstream of δ-hemolysin gene	12.1 (3.2)			10.4 (3.6)	4.0	32.0	3.0	29.9	sa i8854u x at	■	■	■	■	
SACOL2022	*hld*	delta-hemolysin	10.4 (3.8)			6.3 (4.0)	2.3	24.3	1.6	17.1	sa c4144s3498 a at	■	■	■	■	
SACOL2031		ammonium transporter family protein	11.1 (1.3)	5.0 (1.9)	5.6 (1.2)	NC	NC	NC	3.0	2.0	sa c4166s3518 a at	■	■	■	■	
ig SACOL2041-2		intergenic region upstream of SACOL2042 ilvD (ilvD promoter region)	23.4 (4.3)			NC	7.5	NC	24.3	3.5	sa i3561u x at	■	■	■	■	
SACOL2042	*ilvD*	dihydroxy-acid dehydratase	48.5 (1.4)	11.3 (5.4)	26.4 (1.4)	NC	NC	-1.9	13.0	7.0	sa c4209s3561 a at	■	■	■	■	
SACOL2043	*ilvB*	acetolactate synthase large subunit	80.2 (1.2)		39.4 (1.2)	NC	NC	NC	16.0	13.0	sa c4213s3565 a at	■	■	■	■	
SACOL2044		acetolactate synthase 1 regulatory subunit	157.6 (1.7)	8.6 (3.1)	85.9 (1.4)	7.6 (3.1)	4.0	2.3	27.9	13.0	sa c4217s3569 at	■	■	■	■	
ig SACOL2044-5		intergenic region between SACOL2044 and ilvC (2045)			33.7 (1.3)	NC	NC	NC	34.3	NC	sa i8627u x at	■	■	■	■	
SACOL2045	*ilvC*	ketol-acid reductoisomerase	100.4 (1.3)	5.3 (2.2)	61.8 (1.3)	8.4 (2.2)	4.9	3.7	17.1	16.0	sa c9931s8627 a at	■	■	■	■	
SACOL2046	*leuA*	2-isopropylmalate synthase	109.5 (1.2)		64.0 (1.3)	9.5 (2.4)	5.7	3.7	19.7	19.7	sa c4223s3575 a at	■	■	■	■	
SACOL2047	*leuB*	3-isopropylmalate dehydrogenase	89.0 (1.3)	5.2 (2.3)	64.0 (1.4)	8.3 (2.4)	4.6	3.5	21.1	13.9	sa c4225s3576 a at	■	■	■	■	
SACOL2048	*leuC*	isopropylmalate isomerase large subunit	95.3 (1.2)		62.9 (1.4)	7.3 (2.0)	4.0	4.0	13.0	13.9	sa c4229s3580 a at	■	■	■	■	
SACOL2049	*leuD*	isopropylmalate isomerase small subunit	107.6 (1.2)	5.2 (1.7)	76.1 (1.2)	9.0 (1.7)	6.1	5.3	16.0	13.0	sa c4239s3588 a at	■	■	■	■	
SACOL2050	*ilvA2*	threonine dehydratase	78.8 (1.2)	5.2 (1.9)	41.5 (1.2)	5.6 (1.8)	3.5	3.2	11.3	7.5	sa c4243s3594 a at	■	■	■	■	
SACOL2314		sodium/bile acid symporter family protein	7.7 (1.1)			NC	NC	NC	2.1	1.7	sa c5148s4444 a at	■	■	■	■	
SACOL2403		ABC transporter substrate-binding protein	-5.5 (1.4)			-6.7 (1.5)	-6.5	-9.8	-4.0	-8.0	sa c5608s4864 a at	■	■	■	■	
SACOL2554.1		LrgA family protein			-5.0 (1.5)	-2.7 (1.3)	-2.8	-2.0	-3.5	-2.8	sa c6135s5319 a at	■	■	■	■	
SACOL2585		hypothetical protein	20.0 (1.2)	7.7 (2.8)	10.2 (1.5)	NC	NC	NC	4.0	3.0	sa c6262s5443 a at	■	■	■	■	
ig SACOL2585-4		intergenic region downstream of SACOL2585	6.1 (1.1)			NC	NC	NC	NC	NC	sa i5439u x at	■	■	■	■	
SACOL2619		Amino acid permease	19.0 (1.2)	6.6 (2.9)	9.2 (1.7)	NC	NC	-1.5	4.6	3.7	sa c6378s5547 a at	■	■	■	■	
SACOL2620		4-aminobutyrate aminotransferase	29.3 (1.3)	9.0 (3.6)	16.0 (1.5)	NC	NC	-1.6	8.0	4.6	sa c6385s5555 a at	■	■	■	■	
ig SACOL2620-1		intergenic region upstream of SACOL2620	34.3 (1.3)	7.9 (3.4)	20.4 (2.1)	NC	NC	NC	8.0	6.5	sa c10599s11053cv s at	■	■	■	■	
SACOL2627	*betA*	choline dehydrogenase	5.0 (2.4)			NC	-4.9	NC	-6.1	-1.5	sa c9420s8234 a at	■	■	■	■	
SACOL2628	*betB*	betaine aldehyde dehydrogenase	5.4 (2.4)			NC	-4.9	NC	-4.9	NC	sa c6417s5584 a at	■	■	■	■	
SACOL2641	*gpxA2*	Glutathione peroxidase	6.4 (1.4)			NC	NC	NC	3.0	2.3	sa c6467s5637 a at	■	■	■	■	
ig SACOL2641-2		intergenic region between SACOL2642 and gpxA2 (SACOL2641)	8.3 (1.6)			NC	1.7	NC	4.6	2.8	sa i5637u x at	■	■	■	■	
ig_SACOL2641-2 rev comp		reverse complement of intergenic region between SACOL2642 and gpxA2 (SACOL2641)	6.5 (1.8)			NC	NC	NC	NC	NC	sa_i5637ur_x_at	■	■	■	■	
SACOL2659	*aur*	zinc metalloproteinase aureolysin	12.3 (1.6)	5.1 (1.4)	9.2 (1.5)	2.2 (1.3)	1.7	1.9	2.6	2.6	sa c6539s5707 a at	■	■	■	■	
SACOL2689	*icaA*	N-glycosyltransferase	6.7 (1.1)			NC	NC	NC	NC	NC	sa c9442s8255 a at	■	■	■	■	
SACOL2690	*icaD*	intercellular adhesion protein D	7.5 (1.3)			NC	NC	NC	NC	NC	sa c6677s5830 a at	■	■	■	■	
ig SACOL2695-6		intergenic region between SACOL2696 (hisI) and SACOL2695	18.7 (1.5)		6.0 (1.5)	NC	NC	NC	NC	NC	sa i5837u x at	■	■	■	■	
SACOL2696	*hisI*	bifunctional phosphoribosyl-AMP cyclohydrolase/phosphoribosyl-ATP	14.7 (1.2)		11.5 (1.3)	NC	NC	NC	3.2	2.3	sa c9447s10370cs s at	■	■	■	■	
SACOL2697	*hisF*	imidazole glycerol phosphate synthase subunit HisF	16.6 (1.2)		12.3 (1.2)	NC	-1.5	NC	2.1	2.8	sa c6696s10090cs s at	■	■	■	■	
SACOL2698	*hisA*	1-(5-phosphoribosyl)-5-[(5-phosphoribosylamino)methylideneamino] imidazole-4-carboxamide isomerase	16.9 (1.2)		18.4 (1.4)	NC	-2.3	NC	2.5	4.0	sa c6706s5846 a at	■	■	■	■	
SACOL2699	*hisH*	imidazole glycerol phosphate synthase subunit HisH	20.7 (1.1)		14.9 (1.5)	NC	NC	NC	4.9	4.3	sa c6708s5850 a at	■	■	■	■	
SACOL2700	*hisB*	imidazoleglycerol-phosphate dehydratase	22.2 (1.0)		13.9 (1.2)	NC	NC	NC	4.3	4.0	sa c6714s5853 a at	■	■	■	■	
SACOL2701		histidinol-phosphate aminotransferase	24.2 (1.1)		16.9 (1.2)	NC	NC	NC	4.6	4.9	sa c6718s5857 a at	■	■	■	■	
SACOL2702	*hisD*	histidinol dehydrogenase	24.3 (1.1)		14.9 (1.1)	NC	NC	NC	5.3	5.7	sa c6720s5861 a at	■	■	■	■	
SACOL2703	*hisG*	ATP phosphoribosyltransferase catalytic subunit	27.9 (1.1)		11.5 (1.2)	NC	NC	NC	4.9	4.9	sa c6724s5865 a at	■	■	■	■	
SACOL2704	*hisZ*	ATP phosphoribosyltransferase regulatory subunit	23.0 (1.1)		16.9 (1.1)	NC	NC	NC	6.1	6.5	sa c6728s5871 a at	■	■	■	■	
ig SACOL2704-5		intergenic region between SACOL2705-4	11.7 (1.9)		8.7 (1.5)	NC	NC	NC	NC	NC	sa i5871u x at	■	■	■	■	
SACOL2705		hypothetical protein	13.9 (1.3)		10.9 (1.1)	NC	NC	NC	4.3	5.7	sa c6732s5872 a at	■	■	■	■	
ig SACOL2705-6		intergenic region between SACOL2706-5		6.1 (1.8)		NC	NC	NC	NC	NC	sa i5872u x at	■	■	■	■	
SACOL2706		hypothetical protein	18.7 (1.1)		19.4 (1.5)	NC	NC	NC	6.1	7.5	sa c6738s5878 a at	■	■	■	■	
SACOL2707		cobalt transport family protein	20.0 (1.2)		16.9 (1.3)	NC	NC	NC	4.6	5.7	sa c6740s5882 a at	■	■	■	■	
SACOL2708		ABC transporter ATP-binding protein	30.4 (1.3)		19.7 (1.1)	NC	NC	NC	6.1	9.2	sa c5397s4673 a at	■	■	■	■	
SACOL2709		hypothetical protein	31.5 (1.5)		13.5 (1.3)	NC	NC	NC	7.5	6.5	sa c6750s5891 a at	■	■	■	■	
SACOL2710		hypothetical protein	59.7 (1.3)	8.6 (3.6)	24.7 (1.3)	NC	NC	NC	8.0	11.3	sa c6752s5895 a at	■	■	■	■	

Notes:

Best discontinuous megablast or BLASTn hits; for present genes (indicated by black square), probe sequence was present in the genome with at least 97% coverage and at least 96% sequence identity.
